# An ancient ecospecies of *Helicobacter pylori*

**DOI:** 10.1038/s41586-024-07991-z

**Published:** 2024-10-16

**Authors:** Elise Tourrette, Roberto C. Torres, Sarah L. Svensson, Takashi Matsumoto, Muhammad Miftahussurur, Kartika Afrida Fauzia, Ricky Indra Alfaray, Ratha-Korn Vilaichone, Vo Phuoc Tuan, Helicobacter Genomics Consortium, Hafeza Aftab, Lotay Tshering, Dhakal Guru Prasad, Evariste Tshibangu-Kabamba, Ghislain Disashi Tumba, Patrick de Jesus Ngoma-Kisoko, Antoine Tshimpi-Wola, Dieudonné Mumba Ngoyi, Pascal Tshiamala Kashala, Modesto Cruz, José Jiménez Abreu, Celso Hosking, Jukka Ronkainen, Pertti Aro, Titong Sugihartono, Ari Fahrial Syam, Langgeng Agung Waskito, Hasan Maulahela, Yudith Annisa Ayu Rezkitha, Shaho Negahdar Panirani, Hamid Asadzadeh Aghdaei, Mohammad Reza Zali, Nasrin Mirzaei, Saeid Latifi-Navid, Takeshi Matsuhisa, Phawinee Subsomwong, Hideo Terao, Batsaikhan Saruuljavkhlan, Tadashi Shimoyama, Nagisa Kinjo, Fukunori Kinjo, Kazunari Murakami, Thein Myint, Than Than Aye, New Ni, Than Than Yee, Kyaw Htet, Pradeep Krishna Shrestha, Rabi Prakash Sharma, Jeewantha Rathnayake, Meegahalande Durage Lamawansa, Emilio Rudbeck, Lars Agreus, Anna Andreasson, Lars Engstrand, Varocha Mahachai, Thawee Ratanachu-Ek, Kammal Kumar Pawa, Tran Thi Huyen Trang, Tran Thanh Binh, Vu Van Khien, Ho Dang Quy Dung, Dou Narith, Difei Wang, Abbas Yadegar, Lisa M. Olsson, Zhemin Zhou, Yoshio Yamaoka, Kaisa Thorell, Daniel Falush

**Affiliations:** 1 Shanghai Institute of Immunity and Infection, Chinese Academy of Sciences, Shanghai, China; 2 Department of Environmental and Preventive Medicine, Oita University Faculty of Medicine, Yufu, Japan; 3 Universitas Airlangga, Surabaya, Indonesia; 4 Gastroenterology Unit, Department of Medicine and Center of Excellence in Digestive Diseases, Thammasat University, Bangkok, Thailand; 5 Cho Ray Hospital, Ho Chi Minh City, Vietnam; 6 Cancer Genomics Research Lab, Frederick National Lab for Cancer Research, Rockville, MD USA; 7 Foodborne and Waterborne Diseases Research Center, Research Institute for Gastroenterology and Liver Diseases, Shahid Beheshti University of Medical Sciences, Tehran, Iran; 8 The Wallenberg Laboratory, Department of Molecular and Clinical Medicine, Institute of Medicine, Sahlgrenska Academy, University of Gothenburg, Gothenburg, Sweden; 9 Pasteurien College, Suzhou Medical College, Soochow University, Suzhou, China; 10 Department of Medicine, Gastroenterology and Hepatology Section, Baylor College of Medicine, Houston, TX USA; 11 Research center for global and local infectious diseases, Oita University, Yufu, Japan; 12 Department of Chemistry and Molecular Biology, Faculty of Science, University of Gothenburg, Gothenburg, Sweden; 13 Department of Gastroenterology, Dhaka Medical College and Hospital, Dhaka, Bangladesh; 14 Jigme Dorji Wangchuk National Referral Hospital, Thimphu, Bhutan; 15 University of Mbujimayi, Mbujimayi, Democratic Republic of the Congo; 16 University of Kinshasa, Kinshasa, Democratic Republic of the Congo; 17 National Institute of Biomedical Research (INRB), Kinshasa, Democratic Republic of the Congo; 18 Gastroenterology Service, Astryd Clinics, Kinshasa, Democratic Republic of the Congo; 19 Instituto de Microbiología y Parasitología (IMPA), Universidad Autónoma de Santo Domingo, Santo Domingo, Dominican Republic; 20 Universidad Autónoma de Santo Domingo, Santo Domingo, Dominican Republic; 21 Center for Life Course Health Research, University of Oulu, Finland and Primary Health Care Center, Tornio, Finland; 22 Arokero Oy, Tornio, Finland; 23 University of Indonesia, Jakarta, Indonesia; 24 Faculty of Medicine, Muhammadiyah University of Surabaya, Surabaya, Indonesia; 25 Basic and Molecular Epidemiology of Gastrointestinal Disorders Research Center, Research Institute for Gastroenterology and Liver Diseases, Shahid Beheshti University of Medical Sciences, Tehran, Iran; 26 Gastroenterology and Liver Diseases Research Center, Research Institute for Gastroenterology and Liver Diseases, Shahid Beheshti University of Medical Sciences, Tehran, Iran; 27 Department of Biology, Faculty of Sciences, University of Mohaghegh Ardabili, Ardabil, Iran; 28 Nippon Medical School, Tokyo, Japan; 29 Department of Microbiology and Immunology, Hirosaki University Graduate School of Medicine, Hirosaki, Japan; 30 Oita University, Oita, Japan; 31 Aomori General Health Examination Center, Aomori, Japan; 32 Ryusei Hospital, Naha, Japan; 33 Center for Gastroenterology, Urasoe General Hospital, Urasoe, Japan; 34 Department of Gastroenterology, Oita University Faculty of Medicine, Yufu, Japan; 35 Department of Gastroenterology, Yangon General Hospital, University of Medicine, Yangon, Myanmar; 36 Department of Gastroenterology, Thingangyun Sanpya General Hospital, University of Medicine (2), Thingangyun, Myanmar; 37 Department of Gastroenterology, Mandalay General Hospital, University of Medicine, Mandalay, Myanmar; 38 No. 1 Defense Service General Hospital (1000 Bedded), Yangon, Myanmar; 39 Gastroenterology Department, Maharajgunj Medical Campus, Tribhuvan University Hospital, Kathmandu, Nepal; 40 Department of Surgery, Teaching Hospital Peradeniya, University of Peradeniya, Kandy, Sri Lanka; 41 Clinical Genomics Gothenburg, Bioinformatics and Data Centre, University of Gothenburg, Gothenburg, Sweden; 42 Division of Family Medicine, Karolinska Institutet, Stockholm, Sweden; 43 Stress Research Institute, Department of Psychology, Faculty of Social Sciences, Stockholm University, Stockholm, Sweden; 44 Center for Translational Microbiome Research, Department for Microbiology, Tumor and Cell Biology, Karolinska Institutet, Stockholm, Sweden; 45 GI and Liver Center, Bangkok Medical Center, Bangkok, Thailand; 46 Department of Surgery, Rajavithi Hospital, Bangkok, Thailand; 47 Department of Medicine, Chulabhorn International College of Medicine (CICM) at Thammasat University, Pathumthani, Thailand; 48 108 Military Central Hospital, Hanoi, Vietnam; 49 Tam Anh General Hospital, Ho Chi Minh City, Vietnam; 50 Department of Endoscopy, Cho Ray Phnom Penh Hospital, Phnom Penh, Cambodia

**Keywords:** Bacterial evolution, Evolutionary genetics, Bacterial genomics

## Abstract

*Helicobacter pylori* disturbs the stomach lining during long-term colonization of its human host, with sequelae including ulcers and gastric cancer^1,2^. Numerous *H. pylori* virulence factors have been identified, showing extensive geographic variation^1^. Here we identify a ‘Hardy’ ecospecies of *H. pylori* that shares the ancestry of ‘Ubiquitous’ *H. pylori* from the same region in most of the genome but has nearly fixed single-nucleotide polymorphism differences in 100 genes, many of which encode outer membrane proteins and host interaction factors. Most Hardy strains have a second urease, which uses iron as a cofactor rather than nickel^3^, and two additional copies of the vacuolating cytotoxin VacA. Hardy strains currently have a limited distribution, including in Indigenous populations in Siberia and the Americas and in lineages that have jumped from humans to other mammals. Analysis of polymorphism data implies that Hardy and Ubiquitous coexisted in the stomachs of modern humans since before we left Africa and that both were dispersed around the world by our migrations. Our results also show that highly distinct adaptive strategies can arise and be maintained stably within bacterial populations, even in the presence of continuous genetic exchange between strains.

## Abstract (web summary)

‘Hardy’ *Helicobacter* *pylori* ecospecies shares the ancestry of ‘Ubiquitous’ *H. pylori* from the same region in most of the genome but has nearly fixed single-nucleotide polymorphism differences in 100 genes.

## Main

**Extended Data Fig. 1:**
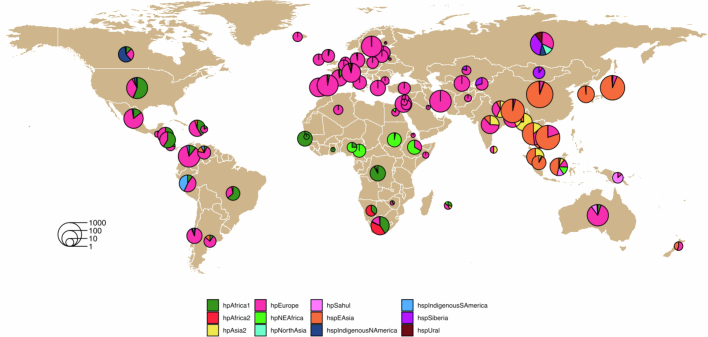
Origin of the different strains of our global dataset. The size of the pie charts shows the number of isolates from the country, with areas scaling logarithmically with sample size. The pie charts show the proportion of isolates assigned to each *H. pylori* population.

To characterize the global diversity of this important human pathogen, we assembled a dataset of 9,188 *Helicobacter* genome sequences (comprising 9,186 *H. pylori* and two *Helicobacter acinonychis*) from humans and other hosts around the world, including from many undersampled populations (Supplementary Tables 1 and 2). Following quality control (see Methods) our subsequent analysis was performed on 6,864 *H. pylori* genomes and two *H. acinonychis*. *H. acinonychis* has been isolated from big cats in zoos and represents a human-to-animal host jump^4^. *H. pylori* has also been occasionally isolated from animals, including four from primates at the UC Davis Primate Research Center, which has housed rhesus macaques and cynomolgus monkeys. In addition to published genomes^5–31^, the dataset contains 2,916 unpublished genomes from 56 countries following quality control, including a large new set of samples from Southeast Asia and Iran, as well as strains previously defined only by multilocus sequence typing, among them a large number of genomes from Siberia. Following previous practice^24,25^, chromosome painting was used to assign the strains to 13 populations (designated hp) and less differentiated subpopulations (designated hsp), each of which have different geographic distributions (Supplementary Table 2 and Extended Data Fig. 1).

## ‘Hardy’ and ‘Ubiquitous’ strains

**Extended Data Fig. 2:**
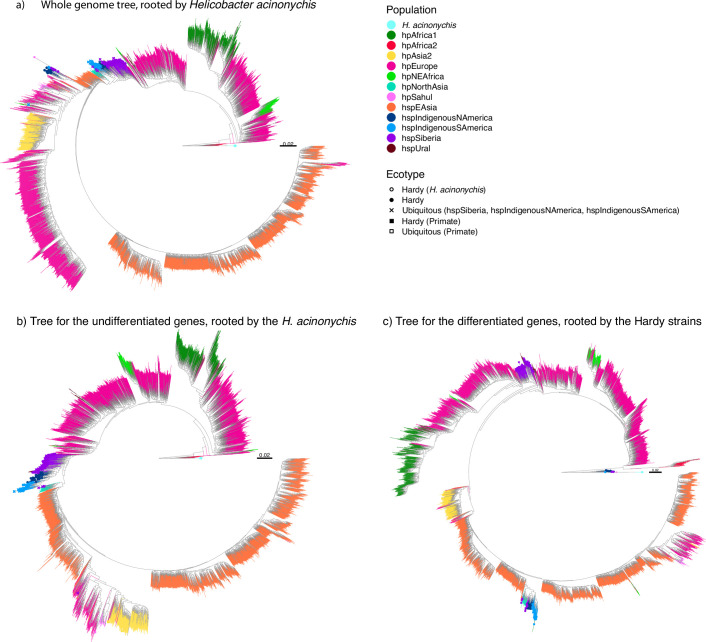
Phylogenetic trees for all strains in the dataset. The branches are coloured based on their population. Hardy strains are represented with a circle while the other strains from the same populations are represented with a cross. The primate strains are represented with squares. Phylogenetic trees for all the genes (A). Some strains from hspIndigenousNAmerica, hspIndigenousSAmerica and hspSiberia do not cluster with their expected population. For undifferentiated genes (B) and for the differentiated genes (C). The branches are coloured based on the population and the strains from the Hardy clade are indicated with a dot.

**Extended Data Fig. 3:**
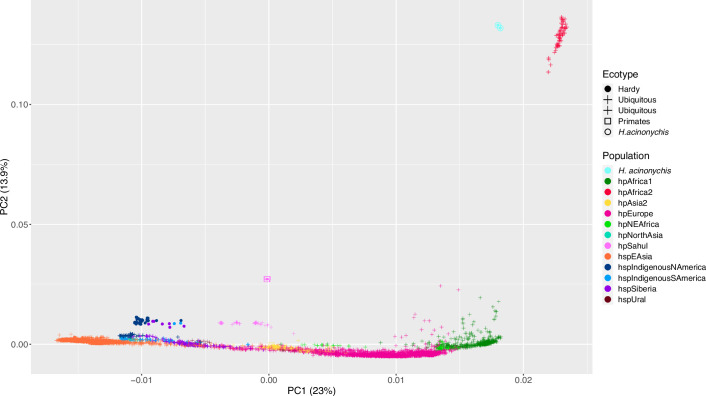
First two components of the Principal Components Analysis (PCA) from the entire dataset. Strains are coloured based on their population and the strains from the Hardy ecospecies are represented by a dot while the crosses represent Ubiquitous strains. Squares and circles respectively indicate primate and *H. acinonychis* strains.

**Extended Data Fig. 4:**
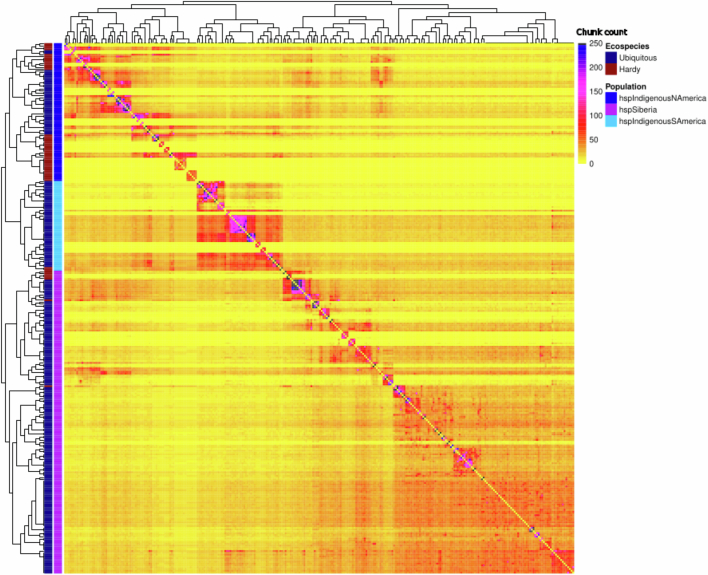
FineSTRUCTURE analysis of the strains from hspSiberia, hspIndigenousNAmerica and hspIndigenousSAmerica. The strains from the Hardy clade are highlighted by red shading, overlaying the dendrogram on the left of the plot, while the Ubiquitous strains are highlighted with blue. FineSTRUCTURE uses an in silico chromosome painting algorithm to fit each strain as a mosaic of nearest neighbours, chosen from the other strains in the dataset. Each row shows the coancestry vector for one strain, which is a count of the number of segments of DNA used in the painting from each of the other strains in the dataset. High coancestry between strains implies that they are nearest neighbours for many segments of the genome and hence share genetic material from a common gene pool. FineSTRUCTURE based clustering is more sensitive to recent gene flow than clustering using genetic distances.

We noticed that we got surprisingly different answers for how strains clustered, depending on the method used (Extended Data Figs. 2a, 3 and 4). A subset of 48 Hardy strains from Chile, Siberia, Canada and the United States, as well as two strains from the UC Davis Primate Center, formed a clade in phylogenetic trees (Extended Data Fig. 2a). The same strains were separated from the Ubiquitous others on a principal components analysis (PCA) plot (Extended Data Fig. 3). Hardy strains were named as such because they were isolated from individuals living in locations that most of the world’s population would consider physically inhospitable, whereas Ubiquitous strains are found in humans everywhere, including the same areas as Hardy. The human Hardy strains were assigned to three different populations, hspSiberia, hspIndigenousNAmerica and hspIndigenousSAmerica, and fineSTRUCTURE^32^ grouped them with strains from their own geographic regions (Extended Data Fig. 4). This unexpected pattern led us to investigate the origin and evolution of these distinct groups, including prediction of the functional consequences of their genomic differences.

**Fig. 1:**
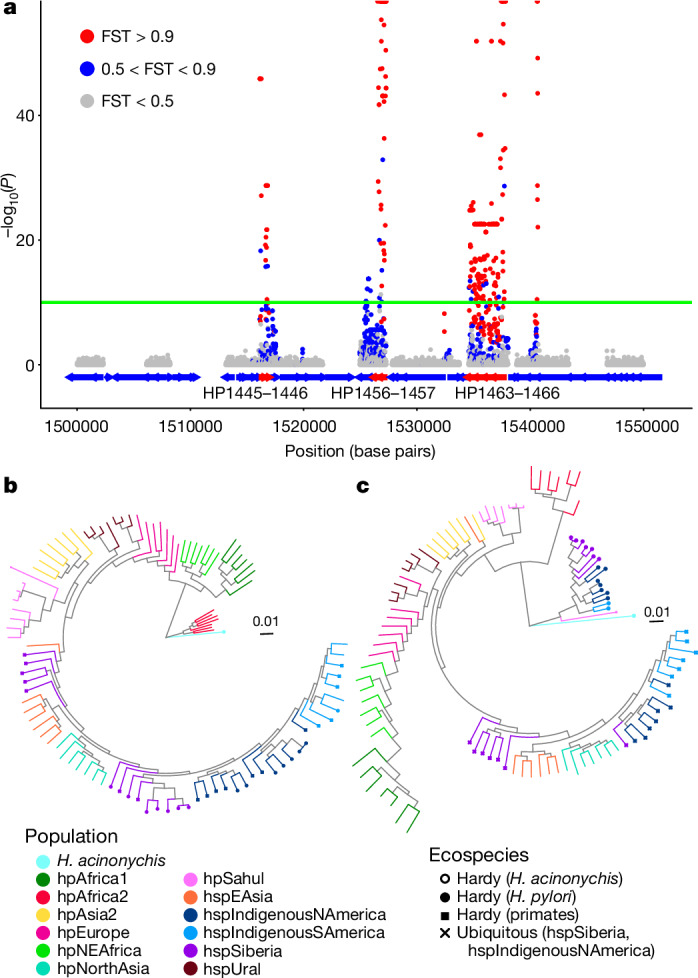
Differentiation between Hardy and Ubiquitous strains is localized in the genome. **a**, Manhattan plot from GWAS analysis of Hardy versus Ubiquitous strains from hspSiberia and hspIndigenousNAmerica (zoomed in between 1.50 and 1.55 megabase pairs; full plot shown in Extended Data Fig. 5). Genes are indicated by blue and red (differentiated genes) arrows. Green line indicates significance threshold (−log_10_(*P*) = 10, which is based on a Bayesian Wald test with a Bonferroni correction for multiple testing, using a significance threshold before correction of 3 × 10^–5^, and 285,792 tested SNPs). Points are coloured based on FST (fixation index) values; half-points at the top of the plot indicate estimated *P* = 0 and FST = 1. HP1445 and so on are *H. pylori* genes based on the annotation of the 26695 strain. **b**,**c**, Phylogenetic trees for undifferentiated (**b**) and differentiated (**c**) genes from a representative subset of strains (see Extended Data Fig. 2b,c for trees of the whole dataset). Branches are coloured based on population. Strains from the Hardy clade are indicated by a filled circle at the end of the branch.

**Extended Data Fig. 5:**
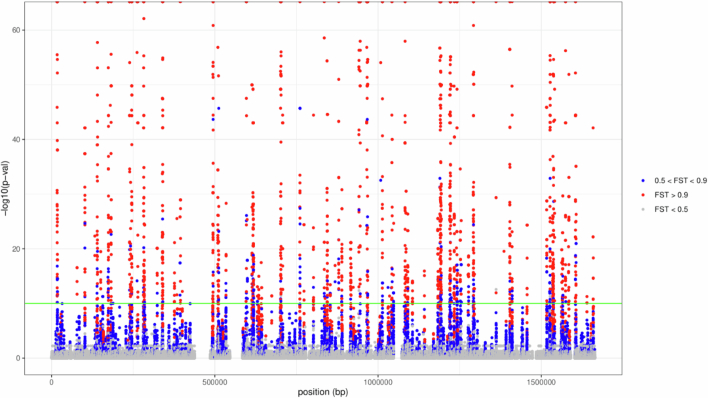
Manhattan plot resulting from a GWAS analysis of the Hardy vs Ubiquitous strains from hspSiberia and hspIndigenousNAmerica. The green line represents the significance threshold (-log10(p) = 10, bayesian Wald test with a correction for multi-testing giving a significance threshold equals to α/n_snps_ = 0.05/285,792). Points are coloured based on FST (fixation index between Hardy and Ubiquitous ecospecies) values (red: FST > 0.9, blue: FST 0.5 − 0.9, grey: FST < 0.5). Half points at the top of the plot indicate an estimated p-value of zero and FST of one.

To understand why strains from different locations and ancestry profiles grouped together by phylogenetic analysis and PCA, we performed genome-wide analysis (genome-wide association study, GWAS) of differentiation between Hardy clade strains and other strains from the same fineSTRUCTURE populations. The differentiation was localized to specific genomic regions and was often confined by gene boundaries (Fig. 1a and Extended Data Fig. 5). We therefore split the genome alignment into genes with nearly fixed differences between the Hardy and Ubiquitous clades (100 of 1,577), genes with no differentiated single-nucleotide polymorphisms (SNPs) (1,034) and intermediate genes that did not fall into the other two categories (443) to characterize patterns of genetic differentiation separately for these fractions of the genome (Supplementary Table 3).

Hardy strains have entirely different genetic relationships with other *H. pylori* at differentiated versus undifferentiated genes, based on phylogenetic trees (Fig. 1b,c and Extended Data Fig. 2b,c). The tree of undifferentiated genes (Fig. 1b and Extended Data Fig. 2b) is consistent with the evolutionary relationships within *H. pylori* established in previous analyses^15,33,34^, with Hardy strains from hspIndigenousSAmerica, hspSiberia and hspIndigenousNAmerica populations clustering with Ubiquitous strains isolated from the same locations. The longest branch in the tree separates strains from the hpAfrica2 population and *H. acinonychis* from other *H. pylori*. Concordant with previous inferences^4,34^, the tree is rooted on this branch. hpAfrica2 strains originated in Khoisan populations in southern Africa^34^, who are the oldest-branching group in the human population tree^35^. By contrast, at differentiated genes (Fig. 1c and Extended Data Fig. 2c), Hardy strains isolated from humans are the most genetically distinct *H. pylori*, branching more deeply than hpAfrica2, and are more closely related to *H. acinonychis*. This divergence causes the Hardy strains to branch separately from Ubiquitous strains from the same population in phylogenetic analyses based on the whole genome (Extended Data Fig. 2a).

**Extended Data Fig. 6:**
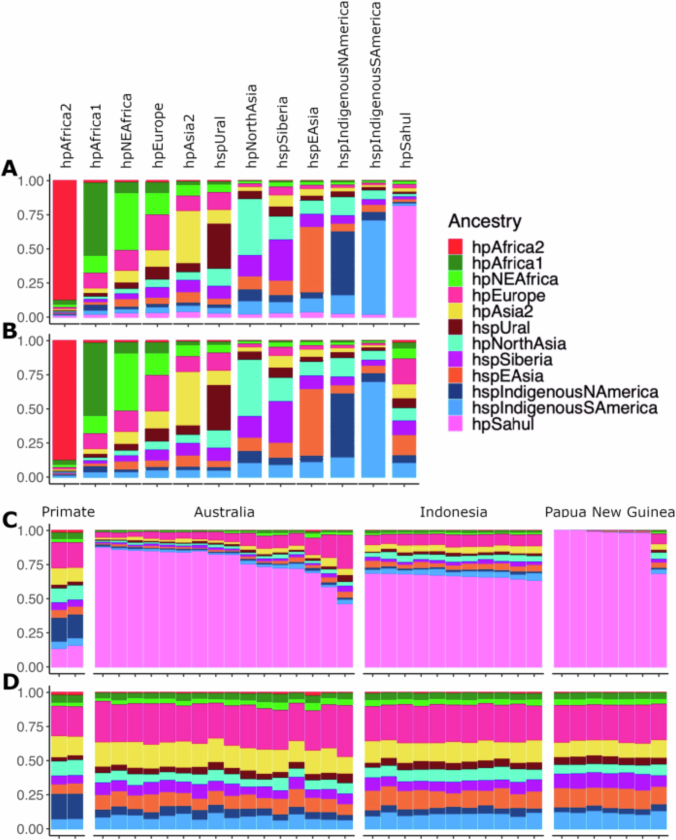
Average ancestry profiles of Global *H. pylori.* (A) With hpSahul donors and (B) without hpSahul donors. Close-up of Hardy primates and hpSahul strains (C) with hpSahul donors and (D) without hpSahul donors. Although the primate strains do not correspond to any hpSahul strains present in the data (based on their ancestry profile in the presence of an hpSahul donor), they can still be assigned to hpSahul (based on their ancestry profile without an hpSahul donor).

The two primate Hardy isolates are assigned to the hpSahul population, found in humans in Indonesia, Papua New Guinea and Australia, and cluster with other isolates from this population in the tree of undifferentiated genes (Fig. 1b and Extended Data Fig. 2b). However, the ancestry profile of these isolates is clearly distinct from that of any other hpSahul isolate in the database (Extended Data Fig. 6), suggesting that the host jump may have been ancient. Two further strains from the UC Davis Primate Research Center belong to the hpAsia2 population (Supplementary Table 2) and appear to be typical Ubiquitous *H. pylori* (data not shown).

## Origin of ecospecies

**Extended Data Fig. 7:**
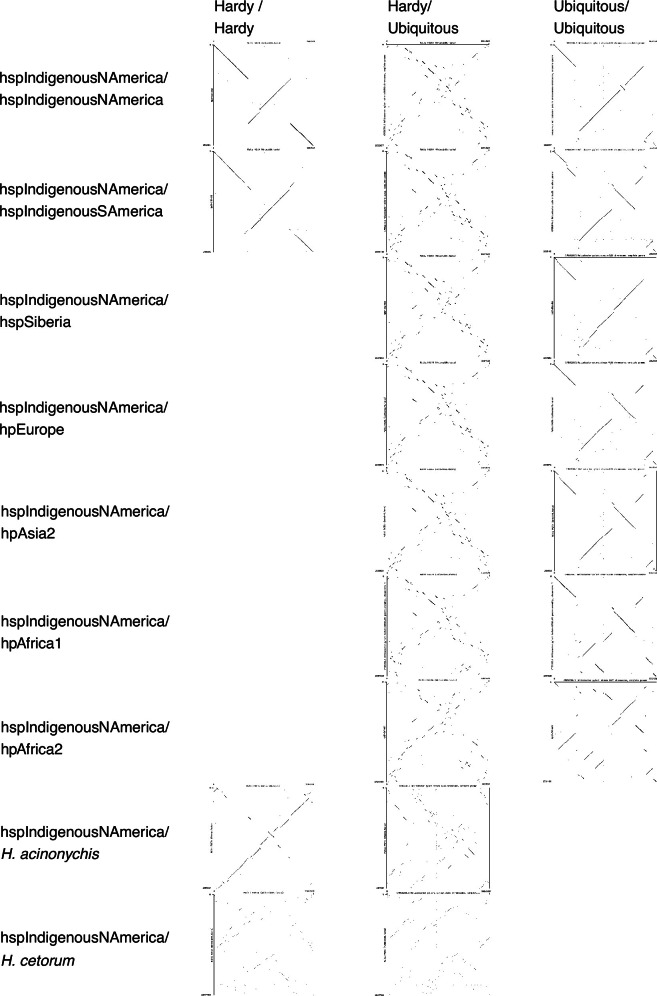
Dot plot comparisons between genomes within and between ecospecies. The genomes of two hspIndigenousNAmerica, one Hardy and one Ubiquitous strain were plotted against the genome of strains more or less distantly related, from left to right: hspIndigenousNAmerica, hspIndigenousSAmerica, hspSiberia, hpAsia2, hpEurope, hpAfrica1, hpAfrica2, *H. acinonychis* and *H. cetorum;* and from top to bottom: Hardy vs Hardy strains, Hardy vs Ubiquitous strains and Ubiquitous vs Ubiquitous strains. Comparison between identical genomes would give single diagonal line, with breaks indicating rearrangements and differences in genome content. The presence of several small lines indicates that there are many rearrangements between the two genomes being compared. On the contrary, comparisons with long lines means highly similar genomes. For more details on how the comparisons were made, see the paragraph “Genome structure comparison” in the Method section.

To understand the global spread of Hardy and Ubiquitous, we next investigated their clonal relationships. Bacteria reproduce by binary fission, meaning that there is a single genealogical tree representing the clonal (cellular) relationships of any sample, but in *H. pylori* homologous recombination is frequent, transferring DNA between strains that coinfect the same host^36^. Recombination of short tracts is unlikely to affect genome order, especially for large rearrangements. Therefore, rearrangements can potentially provide a marker of clonal descent, even in the presence of high recombination rates. Dot plots showed many more rearrangements between any pair of Hardy and Ubiquitous strains than between strains of the same ecospecies (Extended Data Fig. 7), with around 140 rearrangements between two hspIndigenousNAmerica strains from the same ecospecies, compared with around ten within ecospecies^3^. These results show that, at the cellular level, the split between Hardy and Ubiquitous strains was ancient. Rearrangements disrupt homology between strains in the vicinity of breakpoints, but high rates of genetic exchange have continued between ecospecies, probably because of the short length of many fragments incorporated by homologous recombination in *H. pylori*^36^. Nevertheless, at the differentiated genes, Hardy and Ubiquitous strains have retained their distinct identities (Fig. 1c).

**Fig. 2:**
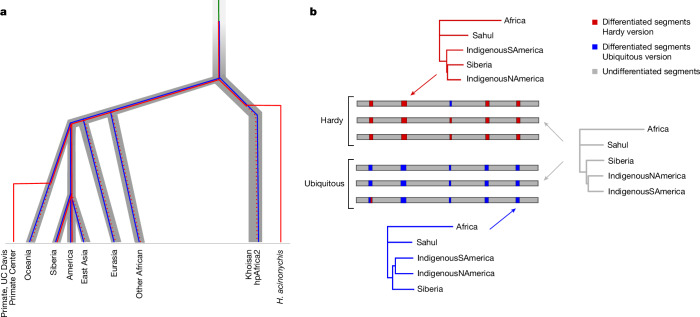
Divergence and spread of Hardy and Ubiquitous gene pools. **a**, Hypothesized scenario for the differentiation of *H. pylori* into two ecospecies and subsequent global spread (not to scale). Thick grey tree represents a simplified history of human population differentiation (based on ref. ^49^). *Helicobacter* evolution is represented by thinner lines, which are within the grey tree during periods of evolution in humans. Green line represents *H. pylori* before the evolution of the two ecospecies; blue line represents Ubiquitous ecospecies; red line represents Hardy ecospecies; dotted red lines indicate that Hardy strains have not yet been detected on the branch and therefore may have gone extinct. **b**, Phylogenetic population trees for differentiated (Hardy in red and Ubiquitous in blue) and undifferentiated (in grey) regions of the genomes. Trees were constructed considering only populations with both Hardy and Ubiquitous representatives. Pairwise distances between strains were calculated for the relevant genome regions, and population distances were calculated by averaging over pairwise strain distances. For Africa, we used *H. acinonychis* strains for the Hardy differentiated gene trees and hpAfrica2 strains for the Ubiquitous differentiated gene tree. For the undifferentiated gene tree, we averaged over the relevant Hardy and Ubiquitous strains.

To reconcile the discordant evolutionary history of differentiated and undifferentiated genes and the ancient split between Hardy and Ubiquitous clonal backgrounds, we hypothesize that *H. pylori* split into two ecospecies before the split of hpAfrica2 from other populations (Fig. 2a). We define ecospecies as bacteria within the same species that have undergone species-level differentiation within a specific fraction of the genome, but otherwise having a single recombining gene pool. It is likely that differentiation in that fraction is maintained by strong ecologically mediated selection against intermediate genotypes. However, other forces, such as genetic mechanisms greatly restricting gene flow in parts of the genome, might also play a role.

We propose that both ecospecies were spread around the world by human migrations. Because the gene pools at differentiated loci are distinct, with each ecospecies having their own segregating SNPs, genetic relationships at these genes represent the separate histories of spread. The DNA may have been carried to their present locations by different human migrations whereas undifferentiated genes reflect an amalgam of both histories. Phylogenetic trees constructed for Hardy and Ubiquitous strains from the same populations at differentiated genes show similar, parallel, histories (Fig. 2b), albeit with minor differences. Specifically, for Ubiquitous and undifferentiated segments, hspIndigenousSAmerica and hspIndigenousNAmerica strains cluster together. In Hardy strains, hspIndigenousSAmerica strains cluster in a deeper position in the tree, hinting that they may have been brought to the Americas by a distinct, and probably earlier, human migration.

**Extended Data Fig. 8:**
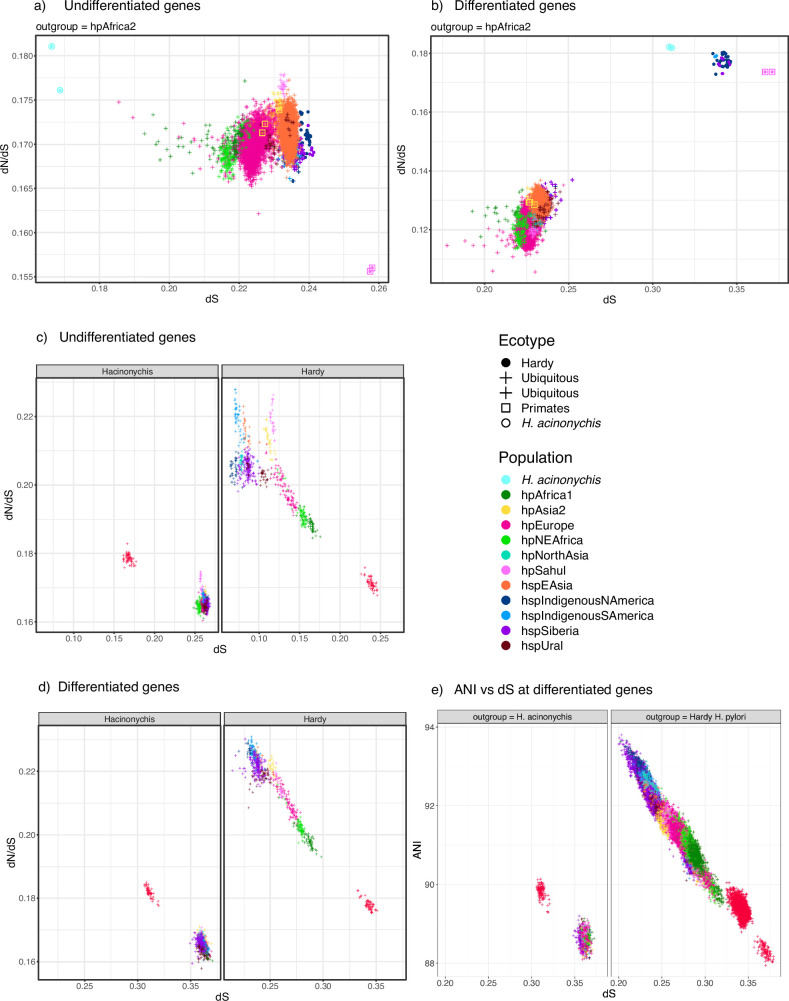
Pairwise dN/dS values to relevant outgroups. dN/dS vs dS (A,B) dN/dS vs dS between the different populations (hpAfrica2 excluded) and hpAfrica2 for the undifferentiated (A) and differentiated (B) genes. Thus, all comparisons involve hpAfrica2 strains and the dots are coloured based on the non-hpAfrica2 population. In addition, the shape represents the ecospecies of the non-hpAfrica2 strain, the dots represent Hardy strains while the crosses represent the Ubiquitous strains; the primates and *H. acinonychis* strains are indicated with squares and circles, respectively. (C,D) dN/dS vs dS between the Ubiquitous and Hardy strains for the undifferentiated (C) and differentiated (D) genes (subplots based on whether the Hardy strains were *H. acinonychis* or non-*H. acinonychis*). For the C and D subplots, all comparisons involve one Hardy (*H. acinonychis* or non-*H. acinonychis*) and one Ubiquitous strain, and the dots are coloured based on the Ubiquitous strain population. In all cases, each dot represents the value for a non-outgroup strain, averaged over their values when compared against the different outgroup strains (the outgroups are hpAfrica2 for subplots A and B and *H. acinonychis* or Hardy *H. pylori* for subplots C and D). (E) Relationship between dS and Average Nucleotide Identity (ANI) for the same comparisons as shown in panel D.

The ratio of non-synonymous to synonymous diversity (dN/dS) indicates the degree of functional constraint on genes, and is also impacted by positive selection (Extended Data Fig. 8a,b). Within Ubiquitous *H. pylori*, dN/dS levels are lower in differentiated genes than in undifferentiated (0.125 versus 0.171), implying that differentiated genes are on average more functionally constrained than the remainder of the genome. However, at differentiated genes, dN/dS levels are elevated in pairwise comparisons involving Hardy and hpAfrica2 strains compared with those in Ubiquitous strains (0.178 versus 0.125). This difference implies that, following differentiation between the two ecospecies, there was an elevated rate of evolution at functional sites at these genes, which is probably associated with specialization to distinct niches.

**Fig. 3:**
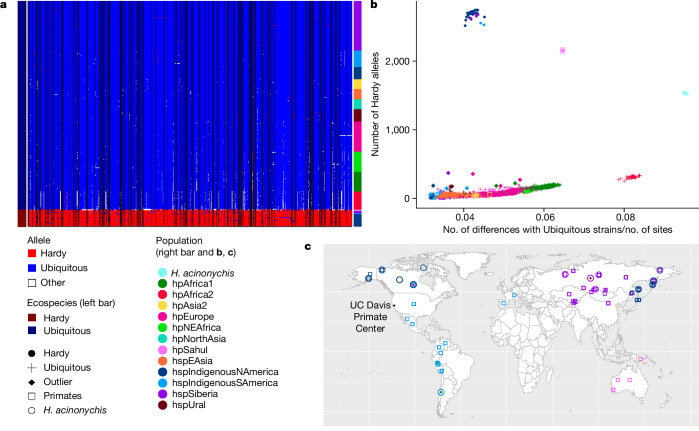
Geographic distribution of Hardy and Ubiquitous haplotypes. **a**, Haplotypes at the SNPs that are differentiated between the two ecospecies for hspSiberia and hspIndigenousNAmerica strains, and randomly selected representatives from other populations. The major Hardy allele is represented in red and the major Ubiquitous allele in blue; other alleles are shown in white. Black vertical lines separate different genes. **b**, Number of Hardy alleles as a function of average genetic distance with Ubiquitous ecospecies strains from hspIndigenousSAmerica, hspSiberia and hspIndigenousNAmerica. Dots are coloured based on their populations with Ubiquitous strains represented by crosses and Hardy strains represented by circles. Filled diamonds indicate Ubiquitous outlier strains with a higher number of Hardy alleles than expected for a strain at that genetic distance. **c**, Map showing location of Hardy (circles) and Ubiquitous (squares) strains from hspIndigenousSAmerica, hspSiberia, hspIndigenousNAmerica and hpSahul populations, as well as strains that were outliers (filled diamonds) compared with their population in the number of Hardy alleles they have. For some strains, information on where they were isolated (latitude and longitude) was missing, in which case we entered the coordinates of their country of isolation.

The fineSTRUCTURE results, with similar levels of coancestry within and between ecospecies in particular geographical locations, demonstrate that there is frequent recombination between ecospecies in most of the genome (Extended Data Fig. 4). The question is, then: what force has maintained the differentiated regions? If this force is purifying selection to retain ecospecies-specific function, then we should see genetic exchange between ecospecies that is too recent to have been purged. The distribution of haplotypes in the differentiated regions fits with the action of purifying selection (Fig. 3a). For many of the differentiated genes there are Hardy strains that have Ubiquitous haplotypes, and vice versa, but each of these haplotypes is present in only one or a small number of strains.

**Extended Data Fig. 9:**
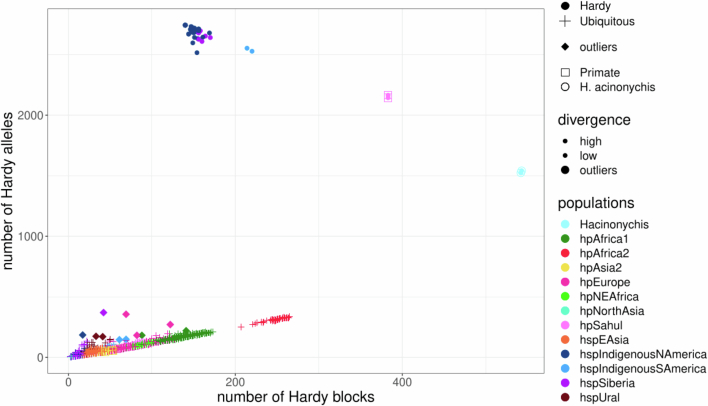
Number of Hardy alleles per strain against the number of Hardy blocks. The dots represent the Hardy strains, and the points are coloured based on their population. The outliers from Fig. 3b are shown with a diamond. The primate and *H. acinonychis* strains are indicated by squares and circles, respectively.

Further evidence that selection has maintained the distinction between ecospecies comes from the limited geographic range of haplotypes originating in the Hardy clade. To identify strains that have received such DNA, we plotted the number of Hardy alleles—that is, the predominant allele in the Hardy clade—as a function of genetic distance to representatives of the Ubiquitous ecospecies from populations in which Hardy ecospecies strains were isolated (Fig. 3b). Distantly related strains (for example, hpAfrica2) have a higher number of such alleles, but this can be attributed to differentiation of the representative Ubiquitous strains rather than to gene flow between ecospecies, as can be seen by the consistent upward trend in Fig. 3b. There are 11 strains (Supplementary Table 2) that are clear outliers from the trend line in Fig. 3b, and all of these derive from geographic regions in which the Hardy ecospecies is found (Fig. 3c). The same outliers appear in a plot of the number of differentiated blocks against the number of differentiated SNPs (Extended Data Fig. 9). Because genetic exchange tends to progressively homogenize ancestry in the absence of natural selection, the absence of outlier strains elsewhere suggests that Hardy alleles are deleterious on a Ubiquitous genetic background and that the outliers themselves can be explained by recent, local genetic exchange. Furthermore, the absence of outliers elsewhere implies that Hardy itself has had a limited geographical range in recent times.

Our hypothesis presented in Fig. 2a implies that Hardy progressively diverged from Ubiquitous by new mutations in the differentiated genes. To investigate evidence for alternative scenarios in which acquisition of DNA from other *Helicobacter* played a role, we assembled a panel of *Helicobacter* species from other mammals, excluding *H. acinonychis* (Supplementary Table 4). We performed BLAST on each of the 100 differentiated genes to identify close matches. In total, 49 genes had significant BLAST matches (Supplementary Table 3), the closest of which was always in *Helicobacter cetorum*, whose hosts are dolphins and whales, and is the closest known relative of *H. pylori* other than *H. acinonychis*.

The results of the BLAST analysis show no evidence for importation of DNA to *H. pylori* from other *Helicobacter* spp. For 29 of the genes, the evolutionary distances, visualized using phylogenetic trees (Supplementary Fig. 1a), were consistent with the scenario shown in Fig. 2a, with Hardy and Ubiquitous versions diverging from each other before the split of hpAfrica2 and *H. acinonychis* from other populations. For 13 of the genes, the tree instead implied that the Hardy and Ubiquitous versions of the gene diverged from each other following the split between hpAfrica2 and other human *H. pylori*, with hpAfrica2 and *H. acinonychis* versions of the gene clustering together (Supplementary Fig. 1b). Three genes (*horL*, hp0599, encoding methyl-accepting chemotaxis protein and *lp220*) showed signs of gene flow between Hardy and Ubiquitous versions (Supplementary Fig. 1c). Three genes, including *vacA* (discussed below), had a history involving gene duplication (Supplementary Fig. 1d) whereas, for the gene encoding the outer membrane protein HopF, the Hardy version diverged from the Ubiquitous version before the split with *H. cetorum* (Supplementary Fig. 1e). This pattern could be explained by cross-species genetic exchange, but an alternative is that the common ancestor of *H. pylori* and *H. cetorum* had two different versions of the gene, with both copies being maintained in *H. pylori* until it split into ecospecies. There was no other gene in which the pattern of polymorphism was suggestive of import of genetic material from other *Helicobacter* species.

## Ecospecies function

**Fig. 4:**
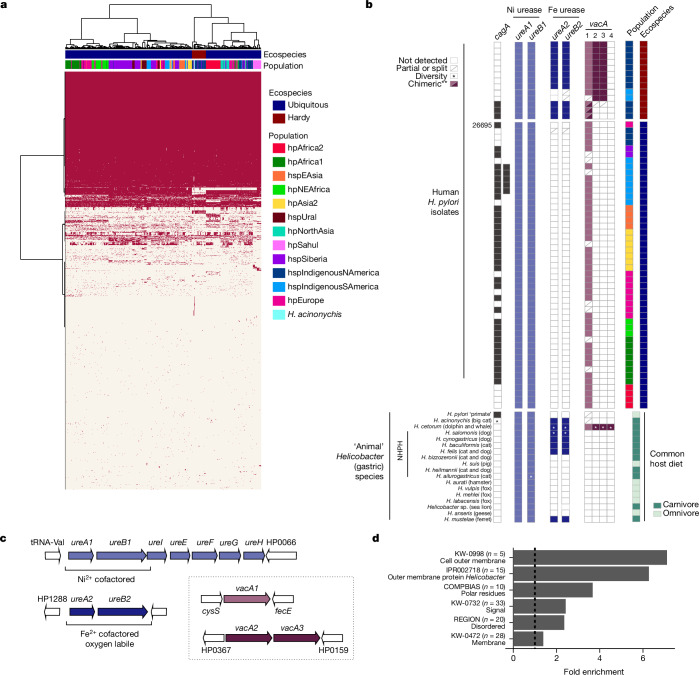
Genome composition in human and animal *Helicobacter.* **a**, Hierarchical clustering based on pangenome presence (red)/absence (white) for a sample of the global dataset. Strains are coloured based on their population and ecospecies. **b**, Top, presence/absence of *cagA* and different *vacA* and *ureA*/*B* types in Hardy and Ubiquitous *H. pylori* from humans. Included is a subset of complete genomes, two *cagA*^+^ Hardy strains and reference strain 26695. Bottom, presence/absence in gastric *Helicobacter* isolated from animals. Common host(s) and their diets are indicated (Supplementary Table 4). **c**, Representative configuration and genomic context of *ureA*/*B* and *vacA* in Hardy *H. pylori* genomes, based on strain HpGP-CAN-006. Lighter-coloured arrows indicate genes present in both ecospecies, based on sequence and genomic context/synteny; darker-coloured arrows indicate Hardy-specific versions. **d**, Fold enrichment for significant (*P* < 0.05, one-sided Fisher’s test, Benjamini correction) functional terms. Chimeric, potential chimeric version (Hardy + Ubiquitous); Diversity, differential presence/absence in analysed genomes within the species; NHPH. non-*H. pylori Helicobacter* spp.; tRNA, transfer RNA.

Clues to the ecological basis of ecospecies divergence are provided by the functions of differentiated and accessory genes from the Hardy ecospecies (Supplementary Table 3). Hardy strains, including *H. acinonychis*, form their own clade in a tree constructed based on accessory genome analysis (Fig. 4a). Only five (the two primate strains included) out of 48 Hardy strains have an intact *cag* pathogenicity island, which is a much lower rate than for the Ubiquitous ecospecies (472 of 673 strains) but sufficient to imply that the island can function and be maintained by natural selection on both ecospecies backgrounds (Fig. 4a,b). The *vacA* gene, coding for vacuolating cytotoxin, is one of the 100 genes differentiated between ecospecies and, in addition, the completely sequenced Hardy genomes show an additional tandem pair of duplicated *vacA* genes (Fig. 4b,c and Supplementary Figs. 1 and 2).

Hardy strains carry an iron-cofactored urease (UreA2B2) in addition to the canonical nickel-dependent enzyme of *H. pylori* (Fig. 4b,c). Iron-cofactored urease is thought to facilitate survival in the low-nickel gastric environment of carnivores^37^. Although previously noted in a single Hardy *H. pylori* strain from northern Canada^3^, this feature has otherwise been entirely associated with *Helicobacter* residing in obligate carnivore animal species: *H. mustelae* (ferrets), *H. felis*/*H. acinonychis* (cats, big cats) and *H. cetorum* (dolphins, whales)^38^. Pangenome analysis of non-*pylori Helicobacter* species isolated from animals (Fig. 4b and Supplementary Table 4) confirmed these associations and identified *ureA2B2* homologues in additional species associated with carnivores (cats, dogs), including *H. cynogastricus*, *H. salomonis* and *H. baculoformis*. We did not detect *ureA2B2* in some gastric *Helicobacter* spp. associated with cats and dogs (for example, *H. heilmannii*, *H. bizzozeronii*); however, these species are known to cocolonize the stomach with other species that carry iron urease such as *H. felis*^39^. We did not detect *ureA2B2* in any species that colonize omnivores, in either the Hardy primate strains that also probably encounter a varied diet (Fig. 4c) or any enterohepatic species (data not shown). The *ureA2B2* locus reflects a very old duplication event, with versions of both genes also found in *H. cetorum* (Supplementary Figs. 3 and 4). Therefore, as we also observed for *vacA*, it is likely that the ancestral population had two versions of the gene but that one has been lost by Ubiquitous strains following evolution of the ecospecies.

Functional enrichment analysis of the 100 differentiated genes in the Hardy ecospecies confirmed a marked enrichment of *Helicobacter* outer membrane proteins (21 of 100 genes, *P* < 0.05; Fig. 4d and Supplementary Tables 3 and 5), and the list also included many genes involved in cell envelope biogenesis. Four outer membrane protein-coding genes are unique to the Hardy genomes. One gene resembles the carbohydrate-binding adhesin BabA, which is missing in Hardy strains, but major alterations in the cysteine loop-forming motifs involved in glycan binding suggest differences in binding specificity.

## Discussion

Our analysis has found that Indigenous people in Siberia and North and South America are infected by two distinct types of *H. pylori* that we have named Hardy and Ubiquitous. These types are distinguished by nearly fixed differences in 100 out of 1,577 genes (based on an alignment with the 26695 reference genome) and important differences in gene content, despite evidence of extensive genetic exchange throughout the genome. We designate these types as ecospecies, because within the differentiated fraction of the genome they have their own gene pools that are sufficiently different (minimum dS of 0.20 between Hardy and Ubiquitous strains and maximum average nucleotide identity (ANI) of 0.94; Extended Data Fig. 8e) that they would be considered a different species based on the usual criteria applied to bacteria of ANI < 0.95 (ref. ^40^), if this divergence was found across the genome. However, they are not species, because most of the genome is undifferentiated and there is no evidence that distinct types ever existed in the undifferentiated fraction of the genome. A similar pattern of genetic variation has been observed in *Vibrio parahaemolyticus*, in which the the EG1a genotype is differentiated at 26 genes in 18 genome regions^41^, despite high recombination elsewhere in the genome^42^, implying that EG1a also deserves ecospecies status.

### Ecospecies evolution in the human stomach

The existence of these highly distinct genotypes within multiple geographical regions poses the question of how and when they have arisen and spread. Our explanation emphasizes continuity within their established primary host species, namely humans. The two ecospecies currently coexist stably within several human populations, and we propose that since the distinction between the types first arose in the ancestor of modern humans the types have coexisted continuously, as shown in Fig. 2a. This hypothesis can explain the current pattern of diversity parsimoniously, without requiring either host jumps into humans^3^ or convergent evolution^43^.

The ecospecies split is ancient. In the regions of the genome in which Hardy and Ubiquitous are distinct, the synonymous divergence between them is higher than that between any pair of strains from the same ecospecies (Extended Data Fig. 8), implying that the Hardy–Ubiquitous split is older than any known *H. pylori* population, including HpAfrica2, which originated in Khoisan groups in southern Africa and is thought to be the deepest-branching human population. Additional evidence for antiquity comes from dot plots showing many more genome rearrangements between pairs of strains in different ecospecies than between pairs within either of them (Extended Data Fig. 7), reflecting an ancient split in their clonal ancestry.

Despite the substantive differences between ecospecies, there is also good evidence of coexistence within the same human populations and parallel spread. First, in most of the genome, coexisting *H. pylori* from different ecospecies are more closely related to each other than they are to *H. pylori* from other geographical locations (Fig. 1b). Second, at the 100 genes that are differentiated between the ecospecies, those from Hardy and Ubiquitous strains have an approximately parallel history of spread, which incorporates Africa, Sahul, Siberia and North and South America, as can be seen from the similar topology and branch length of phylogenetic trees for these loci (Fig. 2b). A simple explanation for this approximately parallel history is that both types have been spread to these continents by the same, or similar, migrations of modern humans, before subsequent host jumps to big cats (*H. acinonychis*) and primates (UC Davis Primate Center isolates). These host jumps took place following the spread of *H. pylori* around the world and can be assigned to specific branches in Fig. 2a, but are probably tens of thousands of years old^4^, because the resulting strains are quite distinct from any *H. pylori* in our sample. The geographical distribution of these lineages in the wild is unknown.

An alternative explanation for the origin of ecospecies is that they arose in an introgression event involving a different *Helicobacter* species or lineage. Our genomic data provide evidence against plausible introgression hypotheses. For example, if humans came out of Africa carrying Ubiquitous *H. pylori* and these hybridized with bacteria with Hardy alleles that had evolved in Neanderthals, Denisovans, big cats or aquatic mammals, this hybridization should have generated genetic exchange throughout the genome, as has been observed wherever distinct *H. pylori* populations coexist^26,33^. However, in the non-differentiated fraction of the genome we see no evidence for elongated branch length in those populations in which the Hardy ecospecies is found (Fig. 1b and Extended Data Fig. 2b) that would be generated by this gene flow, nor evidence of an elevated genetic distance to hpAfrica2 (Extended Data Fig. 8a). If the historical reservoir for Hardy was an archaic human or animal, it is also hard to explain the parallel history of spread shown in Fig. 2b, or indeed how these disparate locations were reached. Few species have been as successful in colonizing the world in the past 100,000 years as modern humans.

Although there is no evidence of import of diverged DNA into *H. pylori* populations, as required by an introgression hypothesis, there is good evidence of progressive divergence at the loci that define the ecospecies. According to the gene-by-gene topologies presented in Supplementary Fig. 1, most of the genes have separate Hardy and Ubiquitous clades. However, for 13 of 49 of these ecospecies-defining genes, hpAfrica2 strains cluster with *H. acinonychis*, implying that the divergence between Hardy and Ubiquitous versions commenced following the split from the hpAfrica2 population—in other words, within the history of extant human populations. Furthermore, there is evidence that positive selection has precipitated divergence, in the form of elevated dN/dS levels in comparisons between ecospecies, at ecospecies-defining genes (Extended Data Fig. 8d).

Notwithstanding the evidence for progressive evolution of Hardy and Ubiquitous ecospecies within humans, important uncertainties remain. The first is the age of the ecospecies split. Previous analysis^34^ suggested that hpAfrica2 split from other *H. pylori* around 100,000 years ago and concluded that there is no direct evidence for *H. pylori* in humans before that. Assuming that divergence in dS values between hpAfrica2 and *H. pylori* Hardy strains at differentiated regions of the genome (Extended Data Fig. 8b,d, Hardy *H. pylori* subplot) accumulated according to a molecular clock implies that Hardy and Ubiquitous evolved at least 0.34/0.23 before this split, or at least 150,000 years ago. However, an alternative hypothesis is that hpAfrica2 strains codiverged with Khoisans, with a split time from other human populations of around 200,000 years—albeit with considerable uncertainty^35,44^. If this is used instead as a calibration point for Hardy evolution, this date estimate is pushed back by a factor of two to 300,000 years. Furthermore, residual gene flow between Hardy and Ubiquitous strains within these differentiated regions could result in substantial underestimation of when divergence between ecospecies commenced. Evidence for such gene flow is that dS values between Hardy and Ubiquitous strains are lowest between strains from the same geographic region (Extended Data Fig. 8d, Hardy *H. pylori* subplot).

### Functional niche of Hardy ecospecies

A second major uncertainty relates to how Hardy strains have been able to reach Sahul, North and South America and Siberia from Africa. One speculative factor that could explain ecospecies distribution is variation in human diets during our colonization history. Hardy strains share an iron-cofactored urease with *Helicobacter* from carnivores. In mammals, the main source of nickel—the cofactor of urease in Ubiquitous strains—is thought to be plant based^37^. This poses a challenge in regard to *Helicobacter* colonizing the stomachs of carnivores. Iron urease is thought to facilitate persistent colonization by species such as *H. felis* and *H. mustelae* even when nickel is limiting^38^. Further evidence for the potential role of diet is that several other genes that are differentiated between Hardy and Ubiquitous strains encode iron and nickel acquisition factors (*frpB4*, *tonB1*, *exbB*, *exbD*) or iron/acid adaptation regulators (*fur*, *arsS*).

Humans themselves have come under selection pressure due to changing diet. Hardy strains are currently found in human populations in which few crops are grown and that have ancestral alleles at the fatty acid desaturase locus^45^, responsible for fatty acid metabolism, that have been strongly selected against within European populations over the past 10,000 years^46^. The modern distribution of *H. pylori* ecospecies could be explained if humans had relied principally on hunting when colonizing new locations but that this depleted large prey^47^, leading to a dietary shift. In this scenario, the first human migrations could have transmitted both ecospecies but Hardy could then have died out in most locations along its migration path, long before the introduction of agriculture. If this scenario is correct, it implies that the ancestral population in which Hardy and Ubiquitous first differentiated was also heavily dependent on hunting. Functional work will be necessary to establish the conditions in which Hardy and Ubiquitous strains are able to coexist, and to further flesh out these hypotheses.

There is an intimate relationship between *H. pylori* pathogenesis and host iron status, and nutritional adaptation has also been proposed as one explanation for the benefit of virulence factors such as VacA and CagA to the bacterium^2^. The Hardy ecospecies has been isolated from human populations with significant gastric disease risk^3^, and *H. acinonychis* causes severe gastritis and is a frequent cause of cheetah death in captivity^48^. However, it remains to be seen whether the Hardy ecospecies can also be defined as a ‘pathotype’. In addition to genes associated with metal uptake, a large fraction of the genes that are differentiated between ecospecies are in outer membrane proteins, including adhesins, with other genes being associated with virulence. This association is fascinating because it could shed light on the interactions among human diet, bacterial colonization strategies and virulence.

## Methods

### Genome collection

We collected a total of 9,188 *Helicobacter* whole-genome sequences from public and private sources, including 4,210 *H. pylori* and one *H. acinonychis* genomes publicly available in Enterobase^50^ (as of 6 June 2022), 1,011 samples from the *Helicobacter pylori* genome project^25^ (10.5281/zenodo.10048320)^51^ and 350 samples available in NCBI, BIGs and Figshare; and 3,615 *H. pylori* novel genomes from different geographic regions around the world and one novel *H. acinonychis*. The novel sequences included 2,133 isolates collected by Y.Y. at the Department of Environmental and Preventive Medicine, Faculty of Medicine, Oita University, Japan, 244 strains from Iran collected by A.Y. and 142 genomes from different parts of the world, including 89 from the Swedish Kalixanda cohort^52^. Lastly, 1,096 worldwide DNA samples and one *H. acinonychis* sample were contributed by Mark Achtman. These sequences have also been deposited in Enterobase at the following workspace: https://enterobase.warwick.ac.uk/a/108555.

Samples from the Yamaoka laboratory were sequenced at Novogene Co., Ltd., Beijing, China with the Illumina NOVA PE150 platform and assembled using the SPAdes genome assembler v.3.15.3 (ref. ^53^) by downsampling read depth to 100 base pairs, specifying a genome size of 1.6 megabase pairs and enabling the option --careful. Of those samples obtained from M.A., 916 were sequenced using either the Illumina MiSeq platform at the University of Gothenburg, Sweden or the Illumina NOVA PE150 platform at Novogene Co., Ltd, UK; a further 180 were sequenced at the University of Warwick, UK. Remaining sequences were sequenced on the Illumina MiSeq platform at Karolinska Institutet and the University of Gothenburg and assembled using the BACTpipe pipeline (10.5281/zenodo.4742358)^54^.

We then filtered out redundant genomes, defined as those sequences with SNP distance below 200, and removed low-quality genomes based on assembly fragmentation (over 500 contigs), coverage to the 26695 *H. pylori* reference strain (below 70%) and contamination (above 90% *H. pylori*) as predicted by Kraken v.2.1.2 (ref. ^55^), to obtain a final total of 6,866 genomes corresponding to the 2,916 genomes first reported in this work and 3,950 that are publicly available (Supplementary Table 2).

### Sequence alignment and core genome variant calling

Single-nucleotide polymorphisms from the core genome (core SNPs) were called using an algorithm based on MUMmer v.3.20 (ref. ^56^), as previously described^57^. We first aligned each genome sequence of the entire dataset to the *H. pylori* 26695 reference strain (NC_000915.1) using nucmer v.3.1. Next, snp-sites v.2.5.2 was used to call all variants from the whole-genome alignment obtained. Variants present in at least 99% of genomes were finally extracted using VCFtools v.0.1.17, generating a total of 866,840 core SNPs.

### Population assignment

To assign a population to the final dataset, we first defined a reference subset of 285 strains consistently assigned into one of 19 *H. pylori* populations/subpopulations in previous reports^15,33,58,59^ and that we confirmed by running fineSTRUCTURE^32^ based on haplotype data from core SNPs, computed for this subset as mentioned above, and using 200,000 iterations of burn-in and Markov chain Monte Carlo. This subset was then considered as a donor panel to paint each sample of the entire dataset using ChromoPainter v.2 (ref. ^32^). Genomes were labelled with a population based on the largest fraction of their genome painted by a population.

### PCA

PCA on the whole dataset was performed using SNPs extracted from the global alignment file, following linkage disequilibrium pruning to remove linked SNPs (window size, 50 base pairs; step size, ten variants; *r*^2^ threshold, 0.1), using the software PLINK (v.1.9)^60^.

The analysis and plotting (for this section and the following) were done using R v.4.3.1 and python v.3.10.6, as well as the R packages ggplot2 v.3.3.6 and tidyverse v.1.3.2 and python library numpy v.1.23.2.

### Phylogenetic analysis

For reconstruction of the various phylogenetic trees, we used coding sequences that were aligned with strain 26695 (see above). When looking at specific genes (*vacA*, *ureA*, *ureB*), gene sequences were first obtained from the individual strains annotation file then aligned using MAFFT (v.7.505, option --auto)^61^. The tree shown in Extended Data Fig. 2a was built using SNPs from all coding sequences. The trees shown in Fig. 1b,c and Extended Data Fig. 2b,c were built using SNPs from the undifferentiated (B panels) and differentiated (C panels) genes, respectively. The trees shown in Supplementary Figs. 1–4 were built using SNPs from specific genes. In addition, for *ureA* (Supplementary Fig. 3) and *ureB* (Supplementary Fig. 4), the sequences were separated into two types because some strains had two copies of the gene. The choice of copy type was based on the similarity between sequences (based on tree clustering and BLAST results, in particular against *H. cetorum*). Using the various alignments of nucleotide sequences, maximum-likelihood trees were constructed using FastTree software^62^ (v.2.1.10, option -nt). The trees were then rooted based on a given outgroup normally used for *H. pylori*: hpAfrica2 and *H. acinonychis* for trees looking at the whole genome or at undifferentiated genes, and Hardy strains for trees looking at differentiated genes. In the case of those trees looking at individual genes, we used *H. cetorum* as an outgroup, these genes having been chosen after their sequences had been BLASTED against the *H. cetorum* genome (see below). Rooting was done using the R package ape^63^ (v.5.7-1, root function). Plotting was done using the R package ggtree v.3.2.1.

The population-level trees shown in Fig. 2b were built via a neighbour-joining algorithm (R package ape, function nj), using a matrix of the average distance between populations represented in both Hardy and Ubiquitous ecospecies. As an equivalent of *H. acinonychis* (Hardy), we used strains from hpAfrica2. Distances between strains were calculated using the dist.dna function (option model, ‘raw’) from the ape R^64^ package. The trees were rooted with hpAfrica2/*H. acinonychis* as outgroups, using the same root function as previously.

### FineSTRUCTURE

To further investigate the population structure of Hardy and Ubiquitous strains, we analysed 295 strains assigned by ChromoPainter to hspIndigenousSAmerica, hspSiberia and hspIndigenousNAmerica *H. pylori* populations by running fineSTRUCTURE with 200,000 iterations of burn-in and Markov chain Monte Carlo, using as input the haplotype data prepared with SNPs from the core genome of those 295 strains, considering only those variants present in more than 99% of the samples.

### GWAS, FST and separation into undifferentiated, intermediate and differentiated genes

Considering the ecospecies as a trait and using the 244 strains from hspSiberia and hspIndigenousNAmerica, we performed GWAS to determine which biallelic core SNPs were significantly associated with the ecospecies. Although Hardy strains were also found in hspIndigenousSAmerica, we chose to remove this population from GWAS analysis due to the small number (two of 49) of Hardy strains. Ubiquitous strains were coded as 0 (198 strains) and Hardy strains as 1 (46 strains). GWAS was performed using the R package bugwas (v.0.0.0.9000)^65^, which considers the population structure using PCA, followed by GEMMA (v.0.93) to perform GWAS analysis. Using a standard significance threshold of −log(*P*) = 5, 4,609 out of 285,792 core biallelic SNPs were significantly associated with the Hardy clade.

To reinforce the results obtained by GWAS, we calculated per-site FST between Ubiquitous and Hardy ecospecies with the R package PopGenome^66^ using the same set of strains and SNPs. We considered a SNP to be differentiated between Hardy and Ubiquitous ecospecies if it was significantly associated with the ecospecies by GWAS (−log(*P*) > 10), and highly differentiated between the two groups based on its FST value (FST > 0.9). Of the core biallelic SNPs we found 2,568 differentiated coding SNPs and 175 differentiated intergenic SNPs. We considered a SNP to be undifferentiated if −log(*P*) < 10 and FS < 0.5 (265,621 coding and 8,950 intergenic SNPs). All other SNPs (7,756 coding and 591 intergenic) were considered intermediate. Following separation of SNPs into three classes, we also distinguished three types of gene based on those present in the 26695 genome: differentiated (100 genes; Supplementary Table 3), containing at least five differentiated SNPs; undifferentiated (1,034 genes), with only undifferentiated SNPs; and the remaining genes (443 genes), which we considered intermediate.

For each strain, we calculated the number of differentiated sites that have the Hardy allele (major allele among the Hardy strains) and compared this number with both the genetic distance to Ubiquitous strains and the number of Hardy blocks. For a given strain, the distance to Ubiquitous strains is the average number of differences between the sequences of this strain and sequences of Ubiquitous strains from hspIndigenousSAmerica, hspSiberia and hspIndigenousNAmerica. The sequences were those aligned on the 26695 sequence, and gaps were removed.

Hardy blocks were defined based on differentiated SNPs: for each strain, if two adjacent differentiated SNPs had the same allele and were part of the same gene, we considered that they were part of the same Hardy block; otherwise, they were from different blocks.

### Pangenome analysis

First, we estimated the gene content of each sample with prokka v.1.4.6 software^67^ using the proteome of 26695 as reference. Then, .gff files were used as input for Panaroo’s v.1.2.8 (ref. ^68^) pangenome pipeline using the strict mode, merging paralogues based on sequence identity of 0.95, length difference of 0.90 and core threshold of 0.95. For this analysis, a smaller dataset was used that consisted of all strains from hspSiberia and hspIndigenousNAmerica (that is, all Hardy strains were included and all Ubiquitous strains from the same populations), as well as randomly chosen strains from the other populations (size of the sample dataset, 721 strains). Then, a hierarchical clustering based on the presence/absence of pangenes was conducted with the pheatmap v.1.0.12 package in R v.4.3.1 using the complete linkage method.

For detection of *cagA*, *vacA* and *ureAB* homologues in diverse *Helicobacter* spp., non-*pylori Helicobacter* genomes were recovered from either GenBank or Enterobase (Supplementary Table 4) and annotated using Prokka. Strains/species were considered if they encoded an intact copy of UreA1B1 (indicating gastric tropism) and available metadata indicated isolation from humans or animals. Metagenome-derived genomes from non-animal hosts were excluded. Host diets were identified using Wikipedia.

For identification of putative homologues, pangenome analysis was performed together with a subset of *H. pylori* strains (Supplementary Table 2) using panaroo with 70% sequence identity and 75% sequence coverage cut-off. For *vacA*, this was supplemented with additional manual inspection (for example, incomplete genomes) using Mauve^69^ and Tablet^70^ and data from the literature (for example, *H. cetorum*^71^).

### Comparison with *H. cetorum* and phylogenetic analysis of differentiated genes

We used *H. cetorum* as an outgroup in the study of differentiated genes, specifically *H. cetorum* strain MIT99-5656 (downloaded 14 February 2023 from NCBI (https://www.ncbi.nlm.nih.gov/data-hub/genome/GCF_000259275.1/). Gene sequences were obtained from the GenBank file provided.

A phylogenetic analysis was performed on differentiated genes, with the *H. cetorum* genome included. First, we obtained *H. cetorum* gene sequences using BLASTing (blastn v.2.11.0)^72^ of a Hardy and a Ubiquitous version of each differentiated gene against the *H. cetorum* genome. For those genes that returned at least one hit, the phylogenetic tree of the gene was generated using FastTree and rooted on the *H. cetorum* sequence (see above).

### Comparison of genome structure

Significant structural variations, including inversions, gaps, repeats and gene cluster rearrangement, are readily visualized using a dot plot. For investigation of genome structure similarity and the difference between Hardy and Ubiquitous groups, we used the Gepard program^73^ (v.1.40 jar file from https://github.com/univieCUBE/gepard) to make the dot plot. Different comparisons were considered: Hardy versus Hardy, Hardy versus Ubiquitous and Ubiquitous versus Ubiquitous and against *H. cetorum*. Publicly available genome sequences were downloaded from NCBI GenBank (https://www.ncbi.nlm.nih.gov/genbank/). The Gepard internal DNA substitution matrix (edna.mat) was selected to generate the alignment and plot. The lower colour limit was 50, to reduce noise and emphasize significant regions. Window size and word length were default values of 0 and 10, respectively.

### Functional enrichment analysis

To determine whether particular types of genes were over-represented among the 100 highly differentiated genes, we performed functional enrichment analysis using the website DAVID^74^. Three hypothetical proteins were removed because they lacked a unique identifier. The background set of genes chosen (see Supplementary Table 3 for a list of genes tested and their categories) for the comparison was based on those genes present in *H. pylori* 26695. A term was considered as significant at *P* < 0.05 following Benjamini correction for multiple tests.

### Calculation of dN/dS and ANI

For each strain in the dataset, we estimated its dN/dS and dS values to an outgroup population using the Yang and Nielsen method (YN00) in PAML (v.4.9) software^75,76^, whereas ANI was calculated with FastANI (v.1.34 (ref. ^77^)). The dN/dS, dS and ANI values shown in plots were averaged over pairwise comparisons with each of the different outgroup strains. Codons that coded for a stop in at least one of the strains were removed from all strains, and dN/dS and ANI values were calculated pairwise using the coding sequences from those aligned to the reference genome (26695, global alignment). In addition, we separated the values between undifferentiated and differentiated coding sequences. We used three different outgroups: hpAfrica2, *H. acinonychis* (Hardy strains) and *H. pylori* Hardy strains, with outgroup population/ecospecies not shown in the plots (only population/ecospecies of the ‘focal’ strain).

### Ethics and inclusion statement

The *Helicobacter* genomics consortium includes gastroenterologists and researchers from several developing countries. Its aim is to characterize the genetic diversity of *Helicobacter* in human populations across the world, and its correlations with gastric disease. In Bangladesh, Bhutan, Congo DR, Dominican Republic, Indonesia, Japan, Myanmar, Nepal, Sri Lanka, Thailand and Vietnam, all preparation for endoscopy surveying was performed by local researchers (persons in the consortium and PhD students at Oita University). Ethical permission for the collection of human gastric biopsy material was obtained for all cohorts, including informed consent from participating individuals. Endoscopy was performed by local physicians and Y.Y. Culturing of bacteria, DNA extraction, next-generation sequencing and basic genetic analysis in these countries were performed at Oita University, principally by doctoral students who were locally recruited in several of the countries and are coauthors of the paper. In addition, the doctors and scientists involved in this consortium are actively involved in the research process and are kept up to date with its findings. This training and dissemination programme will help to spread both genomics knowledge and best practice for treating gastric illness, from Japan, where there has been considerable success in mitigating the burden of gastric cancer and other conditions, to other less developed nations. The study protocol (Iranian strains) was approved by the Institutional Ethical Review Committee of the Research Institute for Gastroenterology and Liver Diseases at Shahid Beheshti University of Medical Sciences, Tehran, Iran (no. IR.SBMU.RIGLD.REC.1395.878). All experiments were performed in accordance with relevant guidelines and regulations recommended by the institution. Written informed consent was obtained from all enroled subjects and/or their legal guardians before sample collection. The sampling of DNA used to generate the new Siberian genomes has been detailed previously^15^. The remaining new genomes were also sourced from previously collected *H. pylori* DNA (for example, refs. ^33,34,59^). The study protocol for the Swedish Kalixanda genomes was approved by Umeå University ethics committee, and the study was conducted in accordance with the Helsinki Declaration. The science of microbiology has not generally viewed human-derived microbes as belonging to the individuals they came from and routine publication of genetic sequences from bacterial pathogens has enabled many public health applications. In a few bacteria, however, *Helicobacter pylori* being one of them, the tight coupling between human and bacterial population structure makes unexpected inferences about human hosts possible from bacterial data, which may be far from the uses envisioned when consent was obtained for sample collection. We will strive to ensure that the design of future studies is built around the needs of communities in which they are performed and that consent procedures provide accurate information on how the samples will be used, informed by recent scientific advances. Our finding of a highly distinct Hardy ecospecies has potential implications for infected individuals in many Indigenous communities known to have a high gastric disease burden. However, the pathogenicity profile, either in single or mixed infection, is currently unknown. In keeping with the TRUST code of conduct, we are maintaining and developing contacts with researchers working in communities where Hardy strains have been isolated with the intention of consulting representatives of these communities to ascertain and accommodate their interests in collaborative research on the functional characterization of these strains, including associations with gastric disease.

### Reporting summary

Further information on research design is available in the Nature Portfolio Reporting Summary linked to this article.

## Online content

Any methods, additional references, Nature Portfolio reporting summaries, source data, extended data, supplementary information, acknowledgements, peer review information; details of author contributions and competing interests; and statements of data and code availability are available at 10.1038/s41586-024-07991-z.

## Supplementary information

Supplementary Figs. This file contains Supplementary Figs. 1–4. Supplementary Fig. 1. Phylogenetic trees of the sequences of differentiated genes that returned at least one hit when BLASTED against the *H. cetorum* genome. Supplementary Fig. 2. Maximum likelihood phylogenetic tree of *vacA*, based on nucleotide sequences from complete *H. pylori* genomes and *H. cetorum*. Supplementary Fig. 3. Phylogenetic tree of *ureA* sequences for a sample of genomes. Supplementary Fig. 4. Phylogenetic tree of *ureB* sequences for a sample of genomes.

Reporting Summary

Supplementary Table 1 List of genomes used and information accessed to retrieve their sequence, accession number, project and references.

Supplementary Table 2 Metadata of the genomes used in the study.

Supplementary Table 3 List of *H. pylori* genes and their metadata, based on annotation of the 26695 reference genome.

Supplementary Table 4 Summary of *cagA*, *vacA* and urease detection in *Helicobacter* strains and species via pangenome analysis.

Supplementary Table 5 Results of functional enrichment analysis. Functional analysis was performed using the DAVID website (Methods). Significance was assessed based on a one-sided Fisher exact test to measure gene enrichment in functional annotation (https://david.ncifcrf.gov/helps/functional_annotation.html#fisher); different corrections for multiple testing (Bonferroni, Benjamini) are also included.

Supplementary Data Data underlying Figs. 1–4 and Supplementary Figs. 1–6 and 8–13.

## Data Availability

The list of strains used is provided in Supplementary Tables 1 and 2, with NCBI accession numbers for all newly sequenced strains given in Supplementary Table 1. The full dataset is also available on the Enterobase worksheet (https://enterobase.warwick.ac.uk/a/108555).

## Code availability

The scripts for the following analyses—PCA, phylogenetic analysis, GWAS, FST and dN/dS—are available at GitHub (https://github.com/EliseTourrette/HpEcospecies_Tourrette2023) and at Zenodo (10.5281/zenodo.12740447)^78^.
