# Supplementary material for: An ancient ecospecies of Helicobacter pylori
Source: Nature. 2024 Oct 16;635(8037):178–85. doi: 10.1038/s41586-024-07991-z (PMC11541087; doi:10.1038/s41586-024-07991-z)
Supplement: Supplementary file 1 — This file contains Supplementary Figs. 1–4. Supplementary Fig. 1. Phylogenetic trees of the sequences of differentiated genes that returned at least one hit when BLASTED against the H. cetorum genome. Supplementary Fig. 2. Maximum likelihood phylogenetic tree of vacA, based on nucleotide sequences from complete H. pylori genomes and H. cetorum. Supplementary Fig. 3. Phylogenetic tree of ureA sequences for a sample of genomes. Supplementary Fig. 4. Phylogenetic tree of ureB sequences for a sample of genomes. [file 41586_2024_7991_MOESM1_ESM.pdf]

nature portfolio

https://doi.org/10.1038/s41586-024-07991-z

Supplementary information

An ancient ecospecies of *Helicobacter pylori*

In the format provided by the authors and unedited

Supplementary Figures

Supplementary Figure 1

Phylogenetic trees of the sequences of differentiated genes that returned at least one hit when blasted against the *H. cetorum* genome. The trees can be separated into four main scenarios: (A) The two ecospecies diverged after the divergence between *H. cetorum* and *H. pylori*, but before the hpAfrica2 branch split (scenario 1). In this scenario, *H. cetorum* is the deepest branch in the tree with the next deepest branch separating Hardy and Ubiquitous strains. The position of the HpAfrica2 branch is variable, likely due to ongoing genetic exchange with other *H. pylori* populations which cooccur in the same human populations in Southern Africa [33]. (B) The divergence takes place after the split with hpAfrica2 (scenario 2). This scenario is similar to (A) except that *H. acinonychis* is the second deepest branch after *H. cetorum*, meaning that human and primate strains cluster together. (C) Presence of gene flow (scenario 3). In this scenario, there are distinct Hardy and Ubiquitous clusters, but some Hardy or Ubiquitous strains have the other type. (D) Variant of scenario 1 but two copies are present. (E) The polymorphism is older than the divergence with *H. cetorum* (scenario 4). In this case, the deepest branch of the tree is between Hardy and Ubiquitous, with *H. cetorum* clustering with one or other.

SupplementaryFigure 2

ML phylogenetic tree of *vacA*, based on nucleotide sequences from complete *H. pylori* genomes and *H. cetorum* (AFI05407.1). The branches are colored based on their population and the Hardy strains are represented with a dot. Contrary to the *vacA* tree that is shown in **Extended Data Fig. 10D**, this tree was built using only complete genomes (in most of the non-complete genomes, *vacA* appears to be highly fragmented due to assembly issues). Only *one* copy of vacA from *H. cetorum* was included as they all clustered on the same branch. The leaves are labeled with strain name, plus the number of the copy of the gene in that strain (i.e., .1, and if there are more than one copy, .2 and .3). Note that, for the Hardy strains, the copies that are closer to the root (generally one copy per Hardy strain) are more *H. pylori*-like than the copies farther away.

Supplementary Figure 3

Phylogenetic tree of *ureA* sequences for a sample of genomes. (A) For all *ureA* copies, (B) for the first copy, which is conserved in all *H. pylori* strains and (C) for the second copy that is only present in the Hardy strains and in some of the Ubiquitous strains from the same populations as the Hardy ones. The strains are colored based on their population, a dot at the end of the branch indicates a Hardy strain. *H. cetorum* sequences are also included.

Supplementary Figure 4

Phylogenetic tree of *ureB* sequences for a sample of genomes. (A) For all *ureB* copies, (B) for the first copy that is present in all strains and (C) for the second copy that is only present in the Hardy strains and in some of the Ubiquitous strains from the same populations as the Hardy ones. The strains are colored based on their population, a dot at the end of the branch indicates a Hardy strain. *H. cetorum* sequences are included.

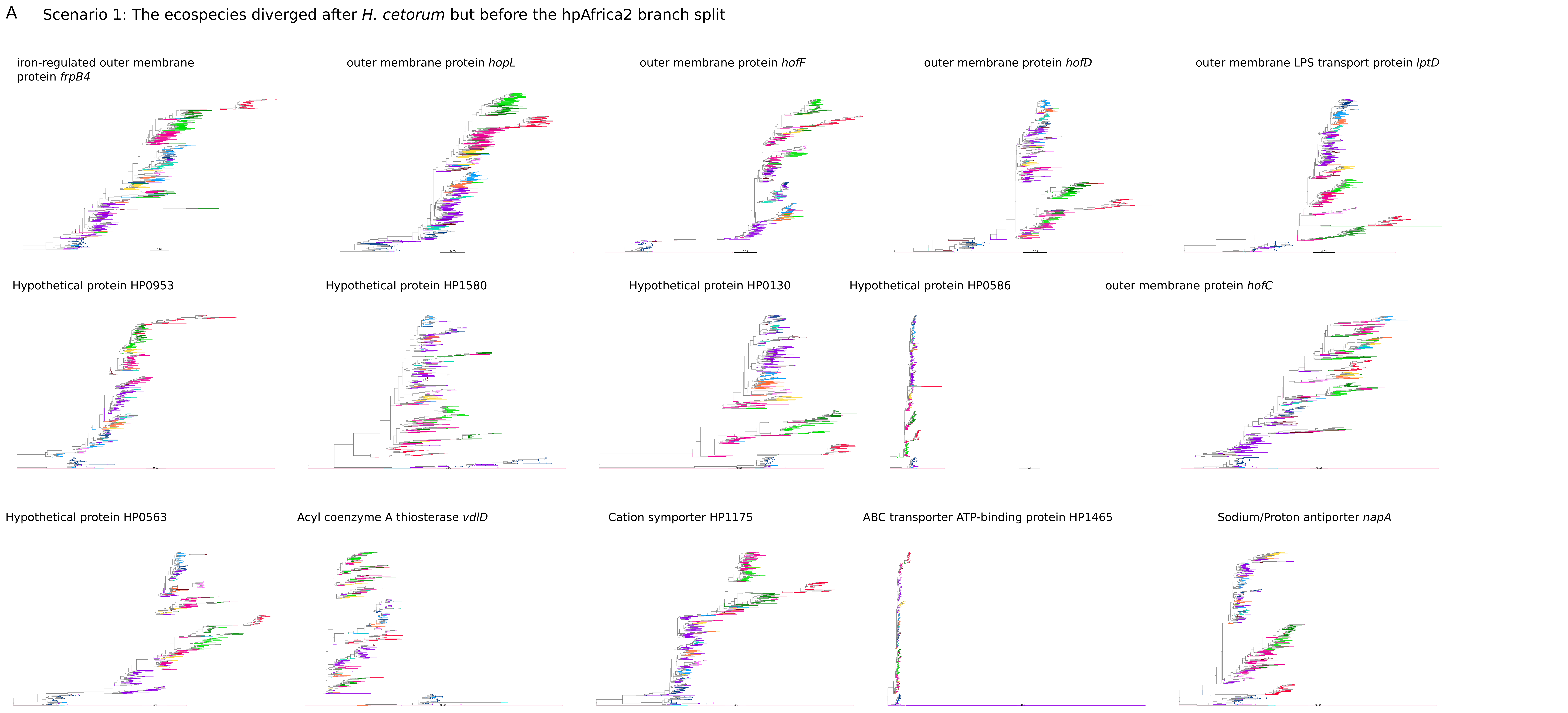
A
Scenario 1: The ecospecies diverged after *H. cetorum* but before the hpAfrica2 branch split
iron-regulated outer membrane protein *frpB4*
outer membrane protein *hopL*
outer membrane protein *hofF*
outer membrane protein *hofD*
outer membrane LPS transport protein *lptD*
Hypothetical protein HP0953
Hypothetical protein HP1580
Hypothetical protein HP0130
Hypothetical protein HP0586
outer membrane protein *hofC*
Hypothetical protein HP0563
Acyl coenzyme A thiosterase *vdlD*
Cation symporter HP1175
ABC transporter ATP-binding protein HP1465
Sodium/Proton antiporter *napA*

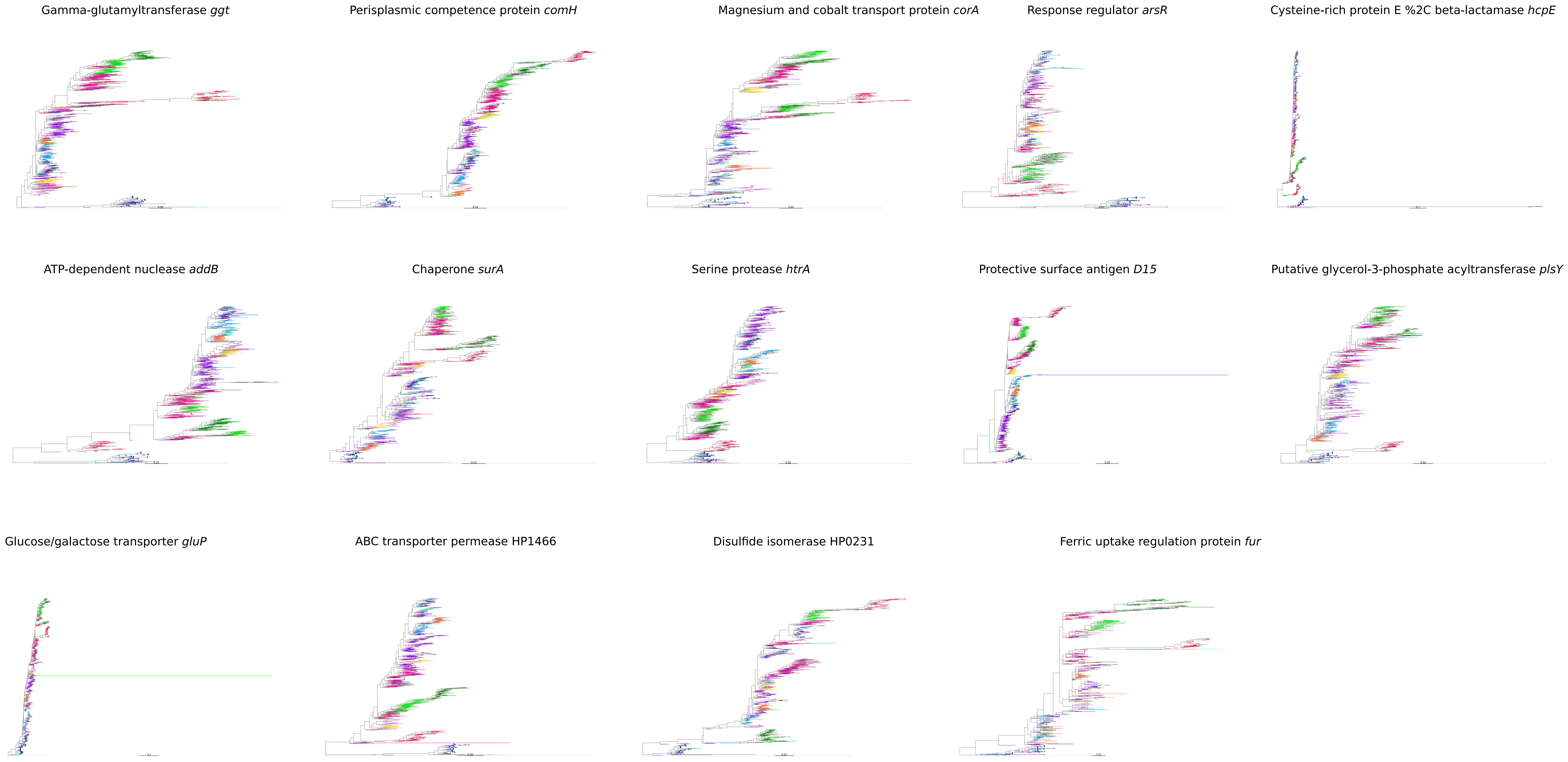
Gamma-glutamyltransferase *ggt*
Perisplasmic competence protein *comH*
Magnesium and cobalt transport protein *corA*
Response regulator *arsR*
Cysteine-rich protein E %2C beta-lactamase *hcpE*
ATP-dependent nuclease *addB*
Chaperone *surA*
Serine protease *htrA*
Protective surface antigen *D15*
Putative glycerol-3-phosphate acyltransferase *plsY*
Glucose/galactose transporter *gluP*
ABC transporter permease HP1466
Disulfide isomerase HP0231
Ferric uptake regulation protein *fur*

B Scenario 2: The divergence takes place after the divergence with hpAfrica2

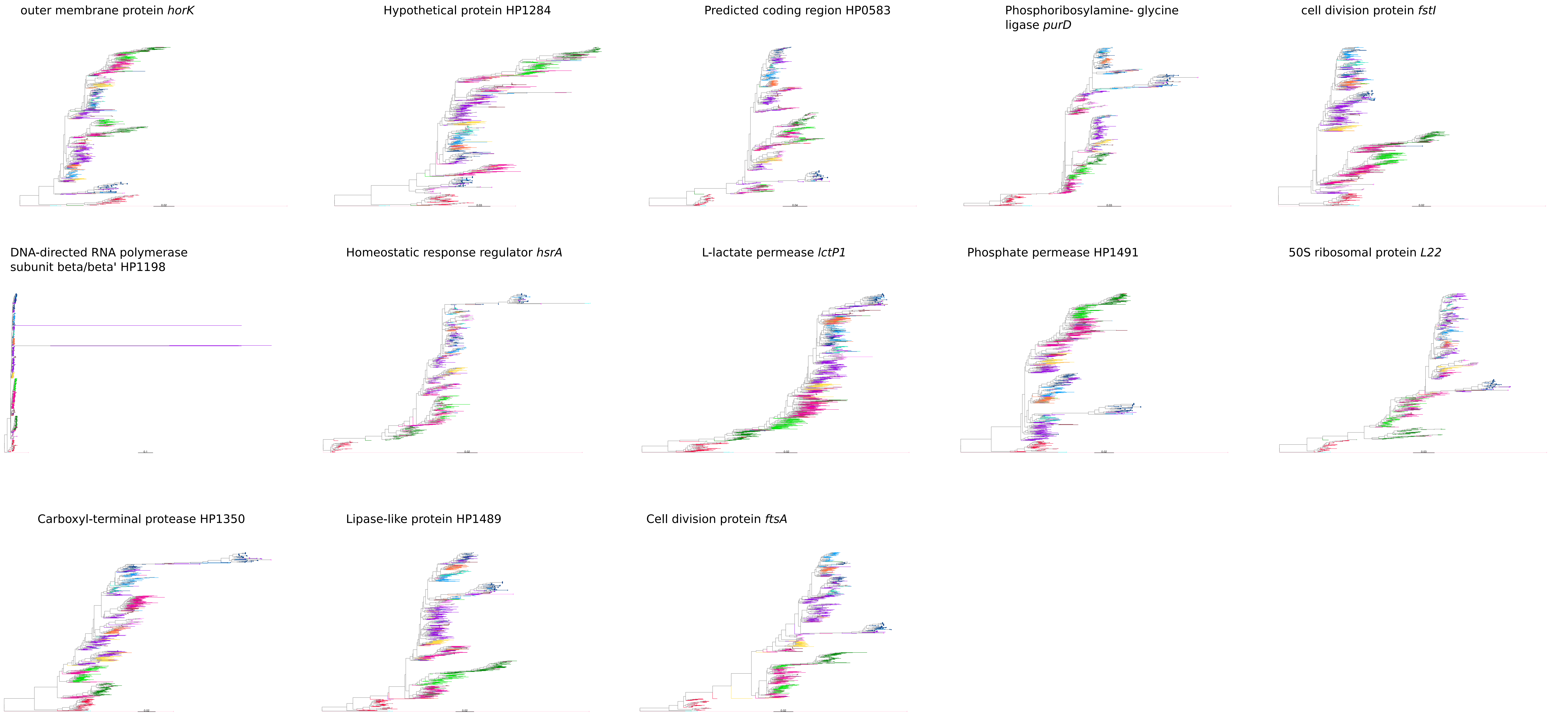

C Scenario 3: Presence of gene flow,in at least part of the gene

outer membrane protein *horL*

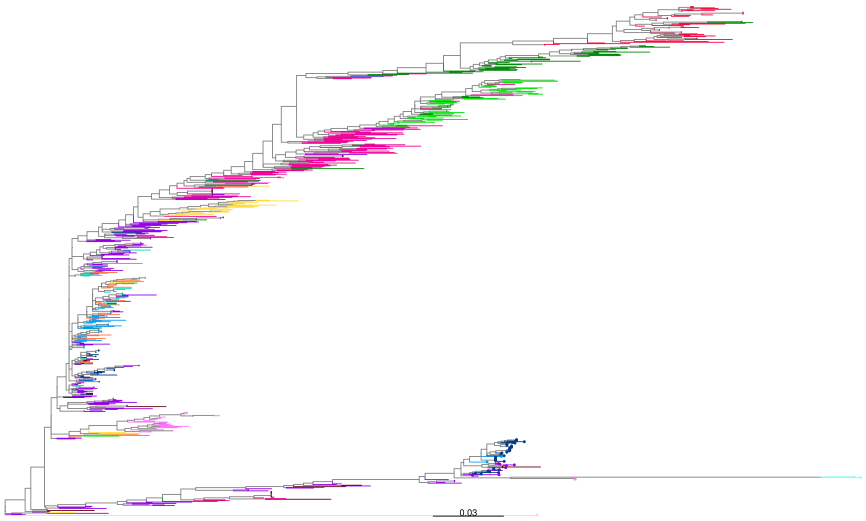

Methyl-accepting chemotaxis protein HP0599

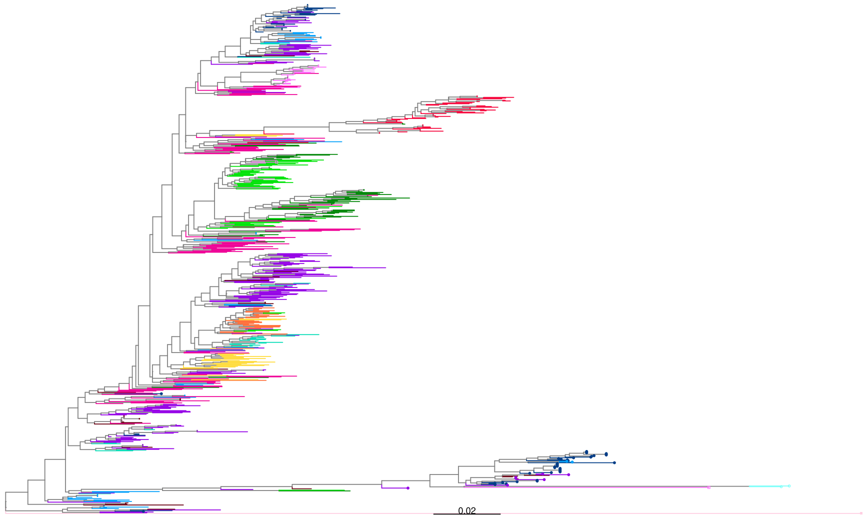

lpp20 lipoprotein

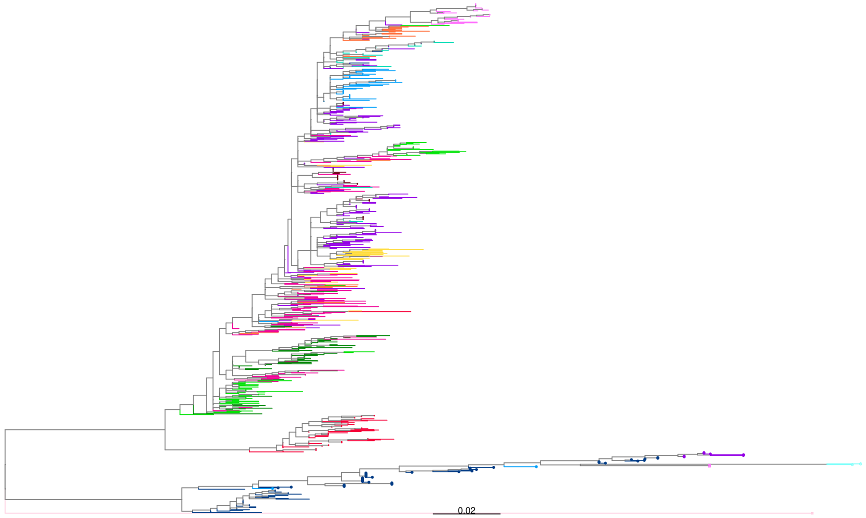

D Scenario 1bis: The ecospecies diverged after *H.cetorum* but before the hpAfrica2 branch split. Two copies are present

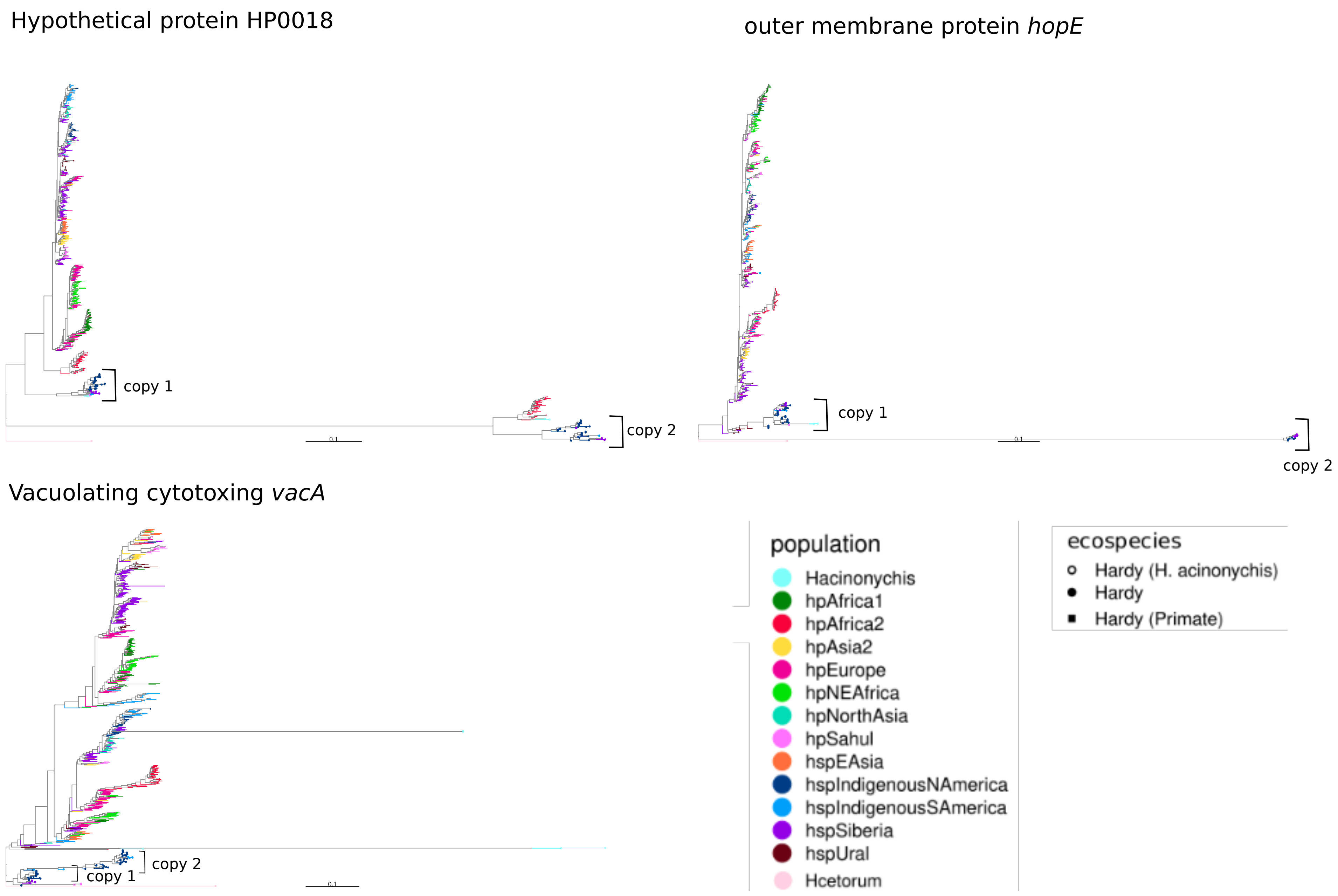

E Scenario 4: The polymorphism is older than *H. cetorum*

outer membrane protein *hopF*

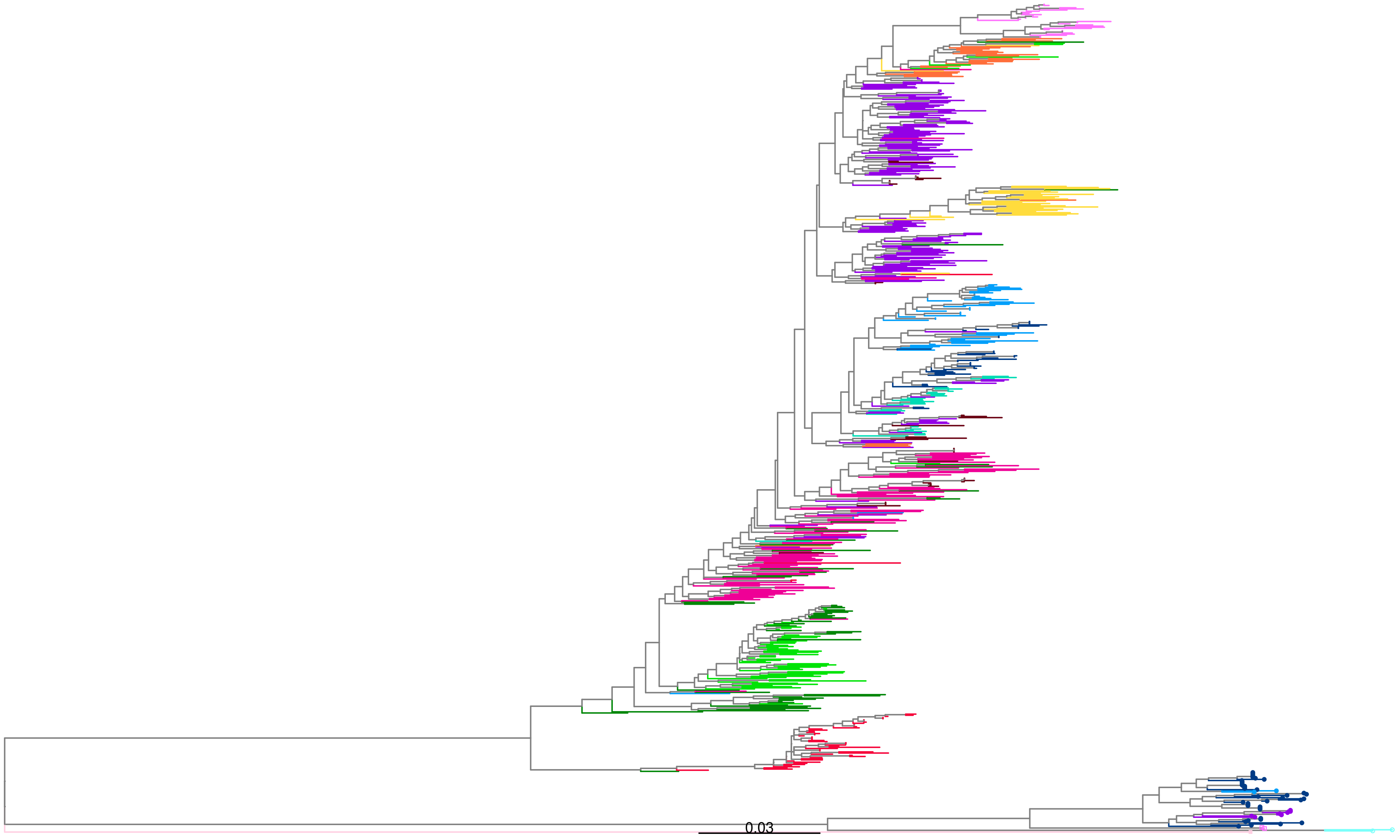

Extended Data Figure 10: **Phylogenetic trees of the sequences of differentiated genes that returned at least one hit when blasted against the *H. cetorum* genome.**

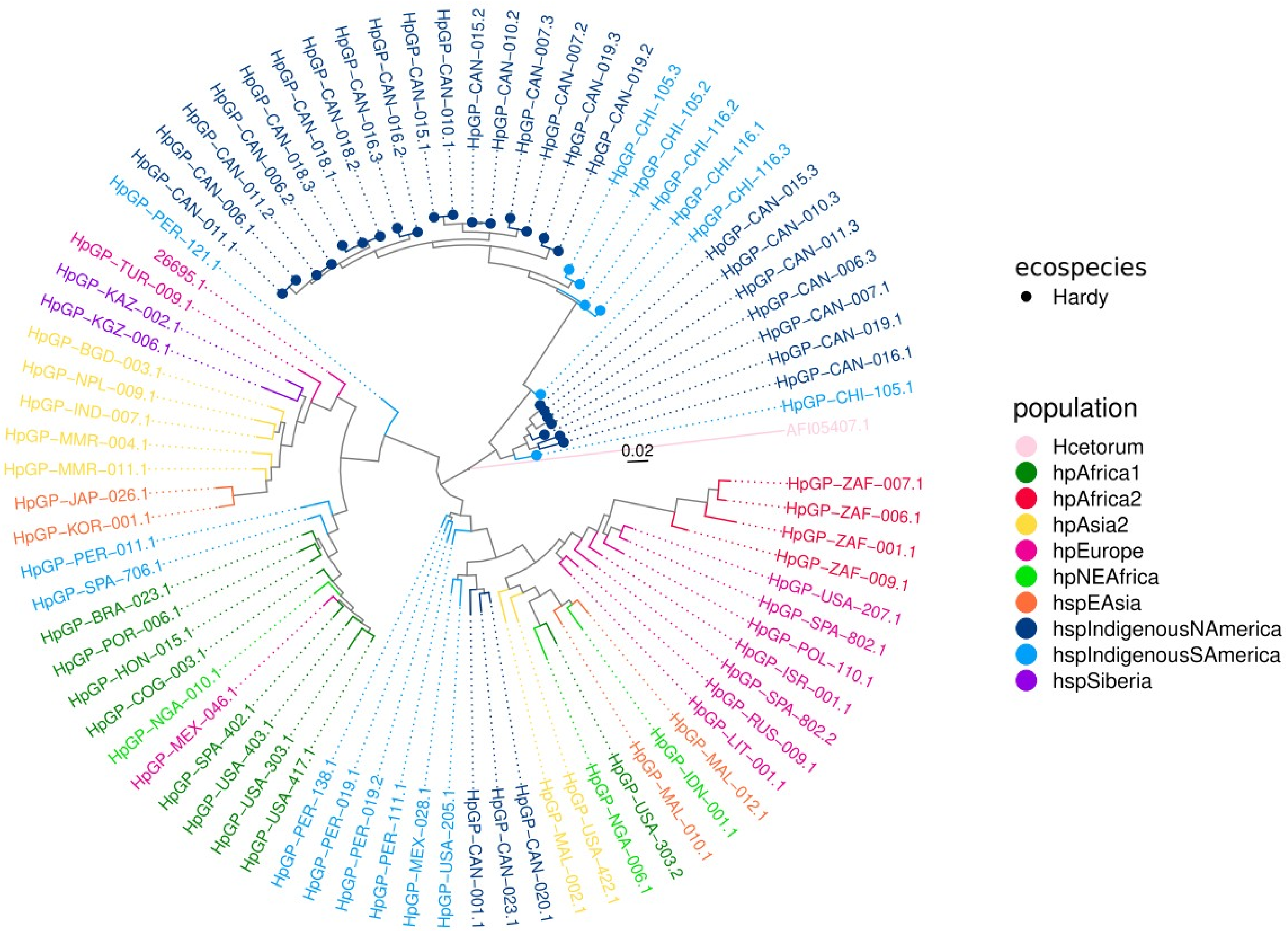

Extended Data Figure 11:

ML phylogenetic tree of *vacA*, based on nucleotide sequences from complete *H. pylori* genomes and *H. cetorum* (AFI05407.1).

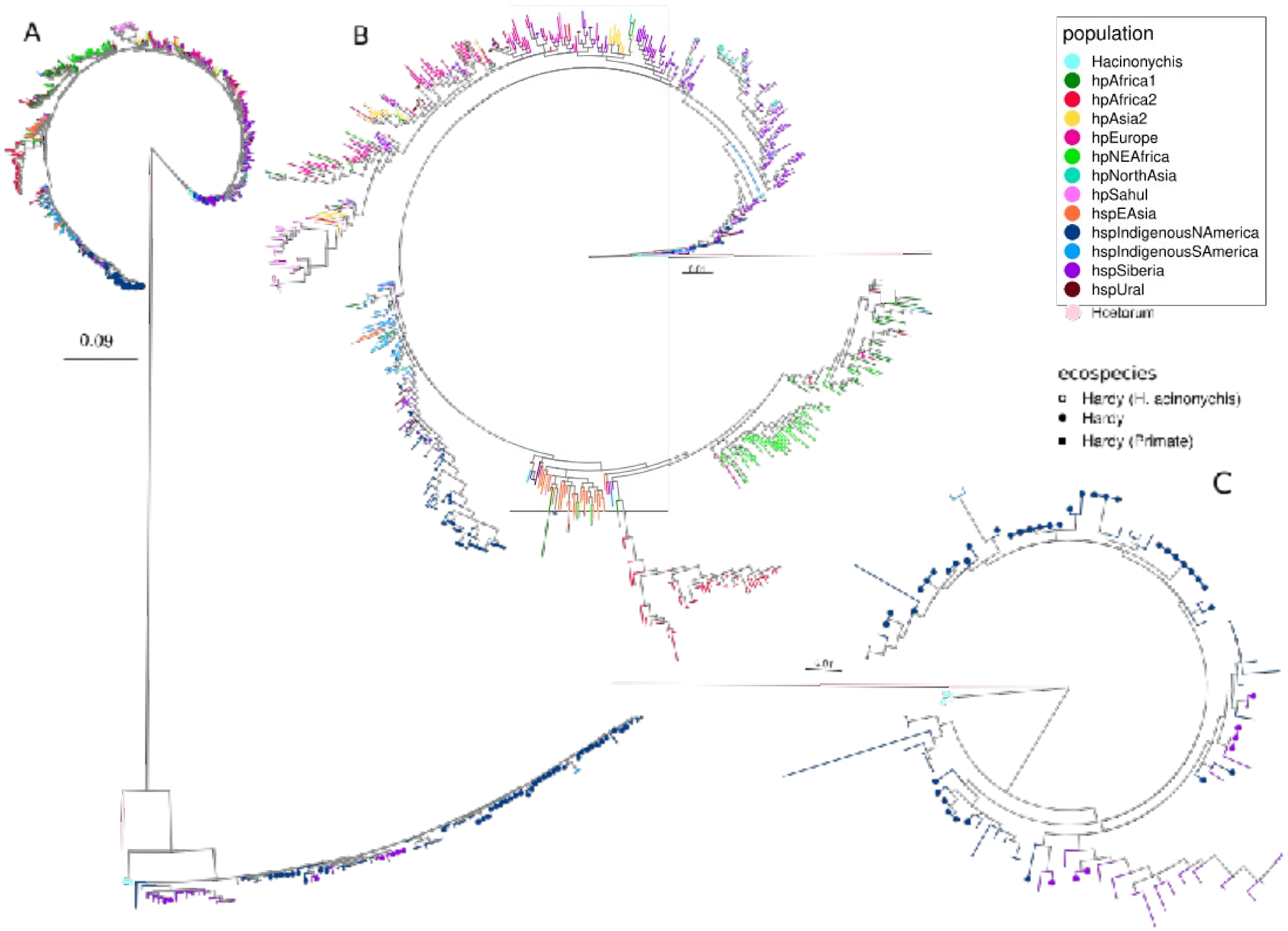

Extended Data Figure 12: **Phylogenetic tree of *ureA* sequences for a sample of strains.**

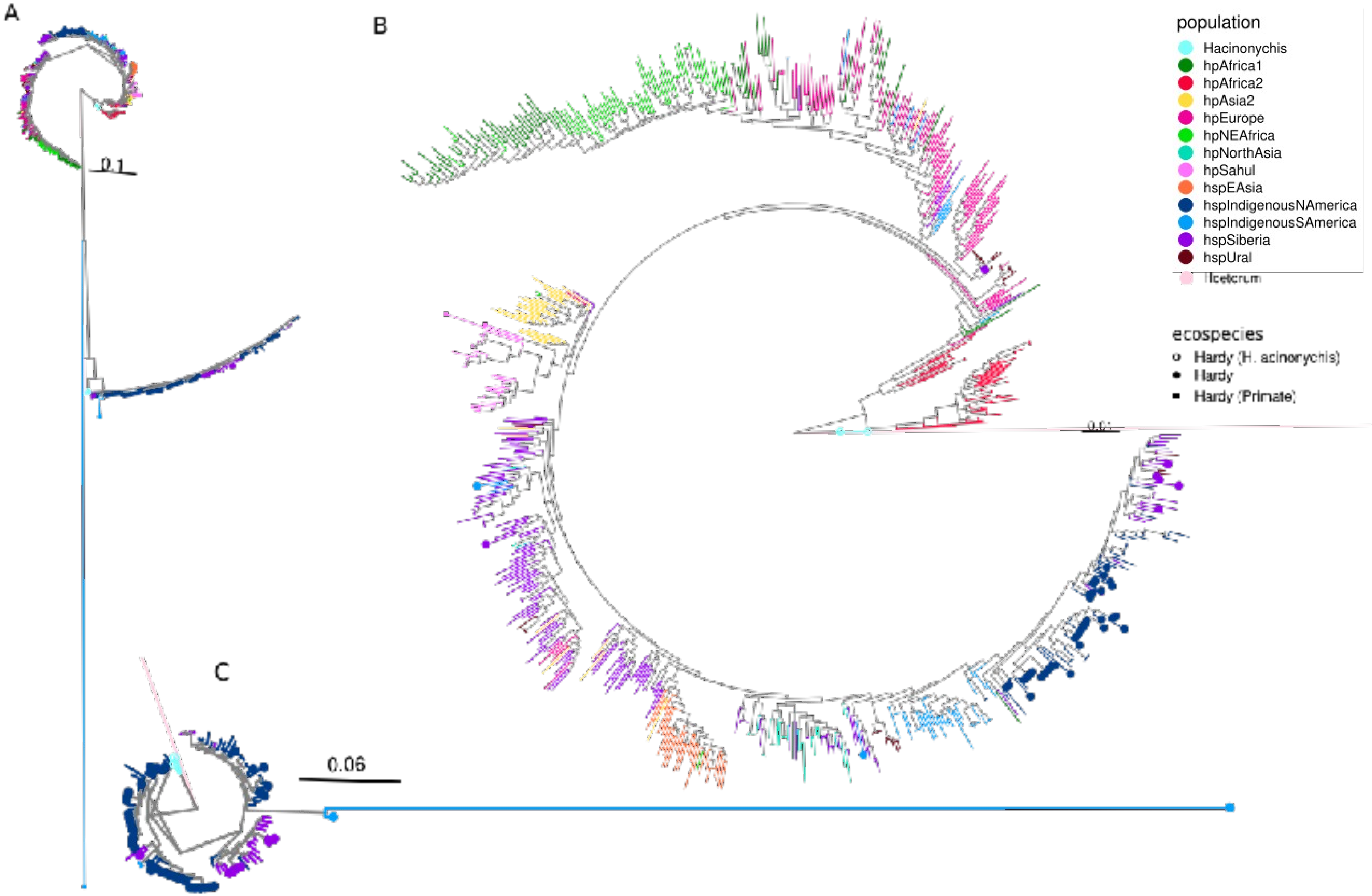

Extended Data Figure 13: **Phylogenetic tree of *ureB* sequences for a sample of strains.**
